# Transcriptome analysis of corpora lutea in domestic cats (*Felis catus*) reveals strong differences in gene expression of various hormones, hormone receptors and regulators across different developmental stages

**DOI:** 10.1186/s12864-025-11510-3

**Published:** 2025-03-31

**Authors:** Beate C. Braun, Michał M. Hryciuk, Dorina Meneghini

**Affiliations:** 1 Department of Reproduction Biology, Leibniz Institute for Zoo and Wildlife Research, 10315 Berlin, Germany; 2 Department for Evolutionary Genetics, Leibniz Institute for Zoo and Wildlife Research, 10315 Berlin, Germany

**Keywords:** Corpus luteum, Domestic Cat, Bulk RNA-Seq, Transcriptome, Adipokines

## Abstract

In the domestic cat (*Felis catus*), the corpus luteum (CL) is the main source of progestogen during pregnancy. Here, we studied gene expression changes in different life cycle stages of the CL of pseudopregnant cats to identify potential regulatory factors. Results revealed no support for different regression substages, which were previously defined on the basis of morphological examination analysis and intraluteal hormone content, as only a very low number of differentially expressed genes and no subclusters in PCA plot were detected. By comparing the regression stage with the developmental/maintenance stage, we detected a total of 6174 differentially expressed genes in the sample set, of which 2882 were upregulated and 3292 were downregulated. The large changes in the expression levels of some genes indicate that the endocrine function of the CL may not be restricted to progesterone (P4) secretion. The findings suggest that domestic cat CLs could also be a source of adipokines such as adiponectin or APELA. The expression of these genes is highly variable and reversed between stages. The life cycle and activity of CLs seem to be regulated by different factors, as genes encoding for the hormone receptors LHCGR and PAQR5 were more highly expressed in the development/maintenance stage, in contrast to this encoding for LEPR, which is higher expressed in regression stage. For regression stage, we identified different potential ways to modulate the cholesterol level and/or P4 concentration. Furthermore, we found differences from previous studies in other species for many genes that were studied in more detail, as well as when analysing functions and pathways. Our findings support the hypothesis that different stages of the CL life cycle in domestic cats can be characterized by changes in gene regulation and that CL life cycles are partly differentially regulated between species.

**Supplementary Information:**

The online version contains supplementary material available at 10.1186/s12864-025-11510-3.

## Background

The corpus luteum (CL) is typically a transient endocrine gland that develops on the side of ovulation on the ovary. Its main function is to produce progestogens, which are crucial for the establishment and maintenance of pregnancy. Usually, progesterone (P4) is the major progestogen. Structurally, corpora lutea (CLs) consist of two different types of steroidogenic cells—small and large luteal cells. These cells arise from the follicular theca and granulosa cells, respectively, following ovulation. In addition, the CL is also composed of a heterogeneous population of nonsteroidogenic cells, including fibroblasts, endothelial cells, pericytes, and various immune cells. The interplay between steroidogenic and nonsteroidogenic cells is crucial for the regulation of luteal lifespan and function. Throughout its lifespan, the CL undergoes continuous changes, progressing through stages of formation, maintenance, and regression. These stages are associated with various biological processes, including cell proliferation, vascularization, luteinization, cell migration, steroidogenesis, and apoptosis [1]. As a result, significant alterations in gene expression occur within the CL during these phases [2].

The domestic cat (*Felis catus*) exhibits a seasonal polyestrous breeding cycle with either induced or spontaneous ovulation [3, 4]. The functional lifespan of the CL (assessed according to the plasma progesterone content published by [5]) is approximately 65 days during pregnancy and approximately 40 days during pseudopregnancy. In domestic cats, the CL is the main source of progesterone during pregnancy [6], but the placenta also produces it without influencing the plasma profile [7]. On the basis of histological observations and intraluteal P4 and estrogen (E) contents [8], the following stages were identified for CL samples collected during pregnancy and pseudopregnancy: formation, development/maintenance (dm), early regression (er), and late regression (lr). Following the pregnant or pseudopregnant luteal phase, the CL transits into the corpus albicans, which may remain visible on the ovary for several months [9]. Previous studies characterizing CLs in domestic cats at different stages of their life cycle have mostly used quantitative real-time qPCR for studying gene expressions. Those studies focused on genes encoding for factors involved in steroidogenesis [6], steroid hormone receptors [10], apoptosis-related factors [11], receptors and the synthesis of prostaglandins [12] and antioxidative enzymes [13]. However, those studies focused on preselected genes, and more studies are needed to unravel previously untargeted genes, e.g., by implementing global gene expression analyses.

Knowledge about the regulation of the CL lifecycle of the domestic cat is important for comparative analysis of the CL lifecycle and functionality within other species, particularly lynxes, which have persistent CLs. Those persistent CLs of lynxes are present on the ovary for a period of at least two years [14] and continue to produce steroids [15]. The domestic cat is a model species for threatened wild felid species. Understanding the CL in domestic cats and other felids is crucial for the successful implementation of assisted reproductive techniques (ART), e.g., luteolysis and estrus induction.

Bulk RNA-Seq offers a powerful approach for transcriptome analysis and exploration of unknown genes involved in physiological pathways [16]. There are a limited number of studies on the transcriptome of CL. These studies have focused primarily on CLs at different stages of the oestrus cycle, e.g., for yaks [2], pigs [17] and dogs [18]; investigated CLs from pregnant and nonpregnant cows [19]; or focused on the effects of various substances, e.g., the influence of prostaglandin F2α (PGF2α) [20].

The aim of this study was to identify potential regulatory factors, such as hormones, receptors, and enzymes, in the CLs of the domestic cat that are expressed depending on the life cycle and could therefore regulate it or have an external regulatory effect. We used a sequence-based approach, bulk RNA-Seq, to investigate the differences in gene expression between CLs in the developmental/maintenance stage (dm) and the regression stage (re) of pseudopregnancy in domestic cats.

## Materials and methods

### Animals and sampling

CL isolation was performed as previously described [21]; 20 samples were collected from 2018 to 2022. Ovaries from pseudopregnant domestic cats were obtained after ovariectomy, performed at animal shelters in Berlin, Germany. Ovariectomies were performed to manage the population of cats and were not related to the purpose of the experiment. The ovaries were transported to the laboratory in HEPES-MEM supplemented with 3 g/L BSA and 1x antibiotic antimycotic solution in 50 mL tubes (Sarstedt AG & Co. KG, Nümbrecht, Germany). Upon arrival, ovaries were isolated from surrounding tissues, washed twice in Dulbecco’s PBS (DPBS), and checked for the presence of CLs. Then, the CLs were isolated and washed with fresh DPBS. After weighing, approximately half of the CL per animal was fixed in Bouin solution and paraffin-embedded for histology. The remaining CL tissues were snap frozen in liquid nitrogen and stored at -80 °C until further use.

### Classification of CL stages

Morphology (HE-stained slices of Bouin-fixed and paraffin-embedded samples) as well as intraluteal hormone content were used to identify the stage of the luteal life cycle. Morphology was assessed as described by Amelkina et al. [8]. To determine the intraluteal hormone content, approximately 5 mg per sample was taken, 200 µl of PBS per 5 mg of tissue was added, and the tissues were homogenized for 2 × 30 s in innuSpeed lysis tube P using a Speed Mill (both Analytik Jena GmbH + Co. KG, Jena, Germany). The lysates were centrifuged (10 min, 10000 × g, 4 °C), and the supernatants were used for extraction. For each sample, 50 µl of lysate supernatant and 450 µl of PBS were transferred to test tubes (16 × 130 mm, Carl Roth GmbH + Co. KG, Karlsruhe, Germany), followed by the addition of 2 ml of methyl-tert-butyl ether/petroleum ether (v: v; 3:7). After shaking for 30 min, the tubes were stored at -80 °C for 15 min. Subsequently, the organic phase was decanted into a new tube (16 × 100 mm, Corning Incorporated, New York, USA). The aqueous phase was extracted a second time as described above for the lysate supernatant with PBS. The organic phases of both extractions were combined and evaporated under a stream of N_2_ for 10 min at 50 °C. Thereafter, the samples were quickly dissolved in 80 µl of 100% methanol and diluted with 120 µl of distilled water. The samples were stored at -20 °C until the hormone content was quantified via ELISA. Progesterone (P4) and estradiol (E2) measurements were carried out with enzyme immunoassays as described earlier [7, 8, 22]. The inter- and intra-assay values for the two biological samples at different concentrations were as follows: 8.4%, 5.1% and 10.4%, 6.7% for P4 and 14.1%, 6.3% and 5.2%, 10.3% for E2, respectively.

### RNA extraction, library preparation, and sequencing

Each tissue sample (~ 5 mg) was homogenized with 350 µl of lysis buffer LBP from the NucleoSpin RNA Plus Kit (Macherey–Nagel GmbH & Co, Düren, Germany) for 2 × 20 s using innuSpeed lysis tubes P and a Speed Mill Plus (both Analytik Jena GmbH + Co. KG, Jena, Germany). After short centrifugation of the homogenized lysate (5 s, 2000 × g), total RNA was extracted from the supernatant following the manufacturer’s instructions. During extraction, genomic DNA was removed by gDNase during on-column DNA digestion. Total RNA sample integrity was measured via Agilent High Sensitivity RNA ScreenTape (Agilent 4150 TapeStation system, Agilent Technologies, Inc., Santa Clara, US). All samples had a RIN value ≥ 7.5. The purified RNA was stored at − 80 °C. Of one microgram of RNA, mRNA was separated with the NEBNext Poly(A) mRNA Magnetic Isolation Module. The cDNA was amplified, and the libraries were generated with the NEBNext Ultra II Directional RNA Library Prep Kit (New England Biolabs GmbH, Frankfurt am Main, Germany) according to the manufacturer’s instructions. For multiplexing during RNA sequencing, NEBNext Multiplex Oligos for Illumina (Dual Index Set 1) were used (New England Biolabs GmbH, Frankfurt am Main, Germany). Libraries were quantified and normalized on the basis of measurements with High Sensitivity D1000 ScreenTape on TapeStation (Agilent, Santa Clara, USA). Sequencing was performed using the Illumina NovaSeq 6000 system (Illumina, San Diego, CA, USA) with 2 × 100 cycles by the Competence Centre for Genomic Analysis (CCGA), Kiel, Germany.

### Read pre-processing, reference genome and mapping to the transcriptome

The raw reads were adapter trimmed using Cutadapt v4.1 with paired-end options [23]. The NEBNext libraries contain adapter sequences similar to Illumina TruSeq libraries (Adaptor sequence read 1: AGATCGGAAGAGCACACGTCTGAACTCCAGTCA, Adaptor sequence read 2: AGATCGGAAGAGCGTCGTGTAGGGAAAGAGTGT). Quality control of adapter-clipped reads was performed by visually checking the results of FastQC v.0.11.9 reports [24], and Preseq 3.1.1 results [25].

To improve the accuracy of transcript quantification, not only the transcript sequences (coding and noncoding) but also the genomic sequences of the *Felis catus* 9 genome, Ensembl release 108: cdna, ncrna, dna.toplevel, and corresponding genome annotation, were used.

Reads were mapped against the reference genome with the selective alignment method from Salmon v1.9.0 [26]. The reference genome was created by concatenating the transcriptome (cdna + ncrna) with genomic sequences using the command ‘salmon index’. The genomic sequences were treated as decoys to identify homologues of known transcriptomic sequences. The transcripts were quantified using ‘salmon quant’ in mapping-based mode with additional parameters: “-l ISR”, “--recoverOrphans“, “--gcBias“, “--seqBias“, “--posBias“, “--thinningFactor 64“, “--minScoreFraction 0.4“, “--softclip“, “--softclipOverhangs”; Out of 893 M paired-end (PE) raw reads, 563 M PE reads could be mapped to the reference genome with average mapping rate of 63% per sample.

### Bulk RNA-seq data analysis

Differential gene expression (DGE) analysis was conducted on raw read counts from Salmon output using DESeq2 R package v1.38.3 [27] in the R statistical environment v4.2.2 [28]. The GTF annotation file of the *Felis catus* 9 genome (Ensembl release 108) was used to create a TxDb database consisting of 54,456 transcripts and 29,550 genes. Using genes that had at least one count, DGE analysis was performed for four stages (dm, er, lr1, lr2) and two stages (dm, re), separately. Genes with an FDR < 0.05 were considered differentially expressed.

By default, DESeq2 assumes that most genes are not differentially expressed, a concept known as the null hypothesis. We applied this null hypothesis with a log2-fold change (lfc) of 0.

For visualization, transformed count data from the variance stabilizing transformation using the function ‘vst’ with the option ‘blind = FALSE’ were used. Heatmaps for sample-to-sample distances, the top 50 most highly variable genes, and significantly differentiated genes with|log2FC| > 7 were generated. Principal component analysis (PCA) plots were created using the top 500 genes with the highest variance across samples, corresponding to the default behaviour of the plotPCA() function from the DESeq2 R package.

The functional enrichment analysis of the DEGs was performed using the Database for Annotation, Visualization, and Integrated Discovery (DAVID) Functional Annotation Tool (FAT), which includes Gene Ontology (GO) analysis and Kyoto Encyclopedia of Genes and Genomes (KEGG) pathway analysis [29, 30]. Using DAVID, the categories “GOTERM_BP_FAT”, “GOTERM_CC_FAT”, “GOTERM_MF_FAT”, and “KEGG_PATHWAY” were selected, and a cut-off threshold of FDR < 0.1 was set. Additionally, the following parameters were used: similarity term overlap = 3, similarity threshold = 0.60, initial group membership = 3, final group membership = 3, multiple linkage threshold = 0.50, and enrichment threshold EASE = 0.1. As input for the “Gene List”, the significantly up- or downregulated genes were used. As input for the “Background List”, pre-filtered genes with at least one count in at least one sample were used. KEGG pathway visualization was performed with the pathview R package v1.38 [31].

### Quantitative real-time PCR

Using the RNA described in RNA Extraction, Library Preparation, and Sequencing, reverse transcription was performed with the PrimeScript RT Reagent Kit (TAKARA BIO INC., Kusatsu, Japan) according to the manufacturer’s manual. Real-time PCR was performed as previously described [7]. The sequences of the primers and annealing temperatures are listed in Table 1. The results obtained for genes of interest were normalized with a normalization factor. This factor was calculated with qbase PLUS software (Biogazelle) [32] on the basis of real-time PCR results for *ACTB*, *GLS* and *KDM4A*.

**Table 1:** Primers and conditions used for quantitative real-time PCR

Gene	Full name	Primer sequence	Product size [bp]	Temperature [◦C]	References
*ACTB*	Actin Beta	fw: GAG CAG GAG ATG GCC ACG rv: CTC GTG GAT GCC ACA GGA	159	62	[15]
*GLS*	Glutaminase	fw: TCC AGC TAT GCT CCA TTG AAG T rv: TGC AGG AAG ACC AAC ATG G	197	61	[7]
*KDM4A*	Lysine Demethylase 4 A	fw: CTA CCA GTG TGA GGT GGT CA rv: CCA TCT GTC CAT CTG ACT TG	168	56.5	This study
*AKR1D1*	Aldo-Keto Reductase Family 1 Member D1	fw: CTT GAA CAA ACC AGG ACT CAA rv: CAG CTT GGA TTC CTT GAG G	153	58.5	This study
*CYP21A2*	Cytochrome P450 Family 21 Subfamily A Member 2	fw: CTG AAG CAG GCC TTG GAG rv: CCT TGG AGC ATG TAG TCG G	113	59.5	This study
*PAQR5*	Progestin And AdipoQ Receptor Family Member 5	fw: CAC ATC TGC TAC TTC CTG GA rv: GAA ACC TGG AGT AGC AGG AG	181	56.5	This study

### Western blot

Protein homogenization and western blot analysis were performed as described previously [6] with the following exceptions. Approximately 10–15 mg of tissue was weighed per sample and mixed with lysis buffer (300 µl per 15 mg). We applied 60 (AKR1D1) and 30 (beta-actin) µg of protein per SDS‒PAGE lane. For transfer, the Trans-Blot Turbo Transfer System of Bio-Rad (Bio-Rad, Herkules, USA) was used (1 A, 25 V, 30 min). The primary antibodies used were mouse anti-AKR1D1 (1:500, sc-36593; Santa Cruz Biotechnology, Inc., Heidelberg, Germany) and mouse anti-beta actin (1:10,000, 8H10010; Novusbio). Chemiluminescence signals were detected with an Azure 600 system (Azure Biosystems, Dublin, USA).

## Results

### CL stages

**Fig. 1:**
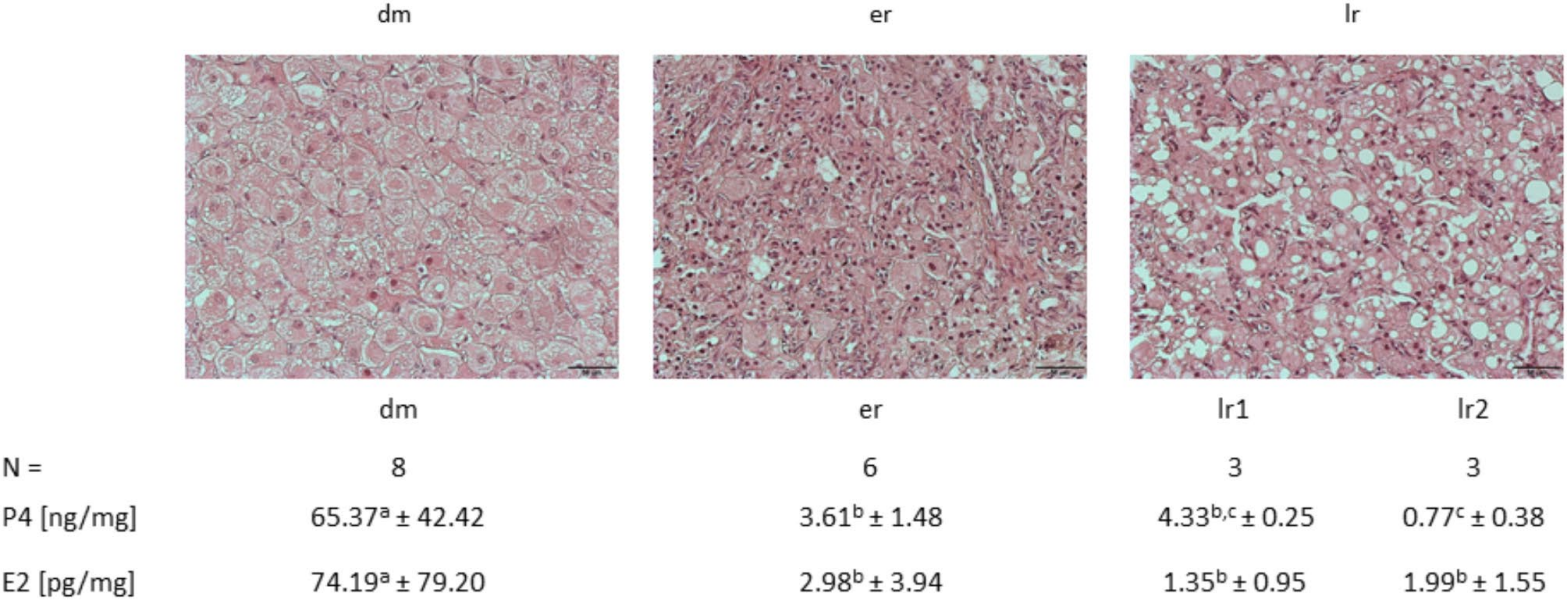
Stages of corpus luteum (CL). HE-stained slides and intraluteal hormone concentrations of progesterone (P4) and estradiol (E2). Statistical significant differences in hormone values are marked by different superscript letters (*p* < 0.05). dm - development/maintenance, er – early regression, lr – late regression (lr1, lr2 – sub-groups of lr)

The morphological assessment resulted in the determination of three groups/stages (dm: *n* = 8; er: *n* = 6, lr: *n* = 6); in addition, the P4 hormone quantification results led to further division of the late regression group into two substages: lr1 and lr2. P4 values in three samples (lr2) were approximately 8-fold lower than those in the remaining three late regression samples (lr1) which had comparable concentrations to er-samples (Fig. 1).

### Gene expression profile and DGE analysis

**Fig. 2:**
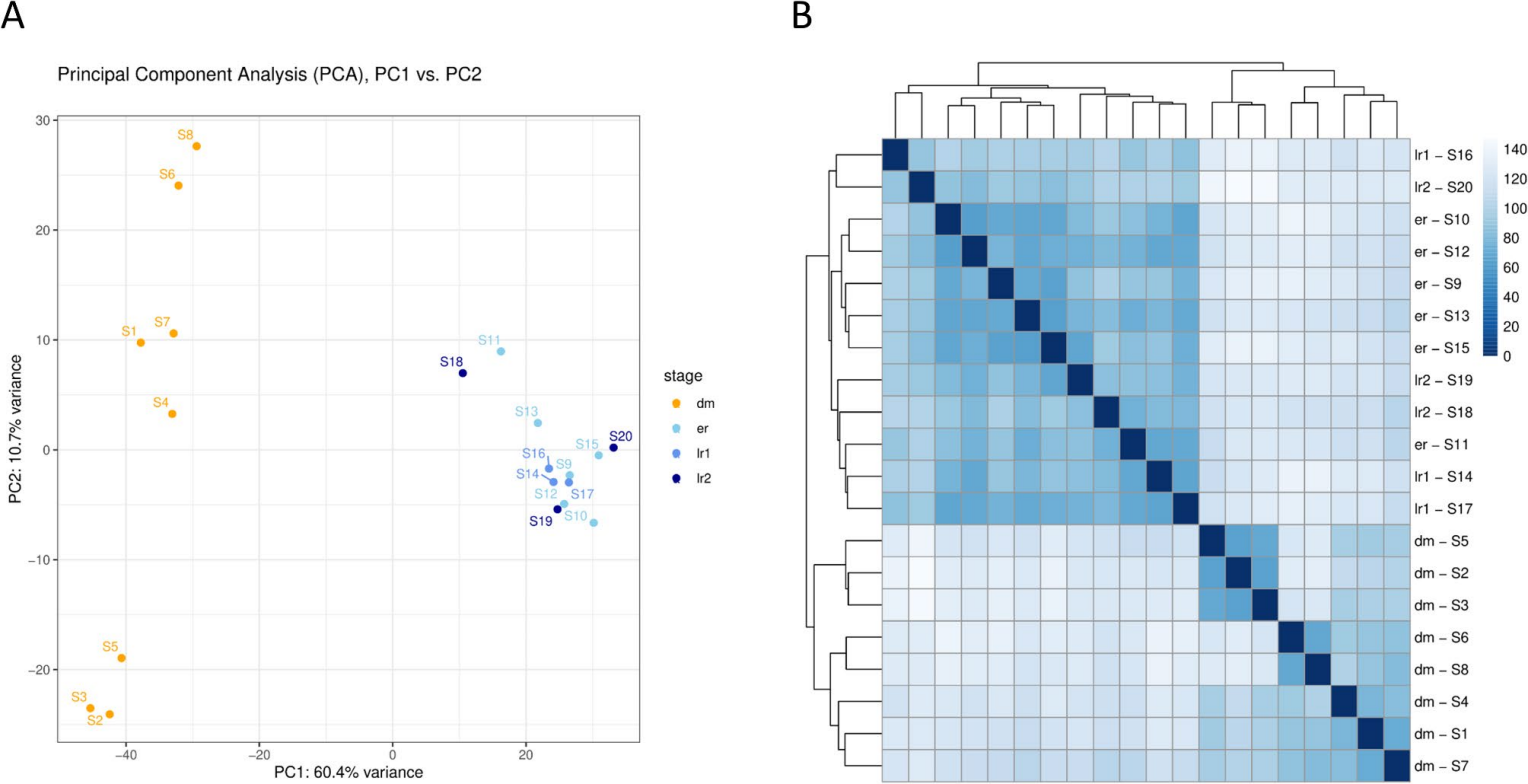
**A**: Principal component analysis plot of 20 samples grouped in four stages. **B**: Heatmap of sample-to-sample distances. dm - development/maintenance, er – early regression, lr –late regression (lr1, lr2 – sub-groups of lr), S- sample

Results of FastQC reports showed a very good read quality per sample after adapter-clipping, with average quality per read was at least 99.9% of reads > = Q20 and at least 96.5% >= Q30 (see Supplemental file 1). Preseq results showed that all sample libraries have a high complexity and are not yet saturated (see current yield and expected future yield of distinct reads in tab “Preseq results” in Supplemental file 1). Overall, we identified 21,701 genes with at least one transcript in at least one sample in our dataset of 20 samples (Supplemental file 2). The results of the PCA plots suggested that there were no clear subclusters for the three regression stages we defined before as early regression (er) and late regressions 1 and 2 (lr1, lr2) (Fig. 2A). Heatmap analysis of sample-to-sample distances also revealed that the predefined regression stages were not detectable as distinct groups (Fig. 2B). Additionally, only a very small number of differentially expressed genes (DEGs) could be detected by comparing the different regression stages (Table 2).

**Table 2:** Number of differentially expressed genes (DEGs)

Contrast of stages	Up-regulated	Down-regulated	Sum of regulated
er vs. dm	2347	2942	5289
lr1 vs. dm	1607	2033	3640
lr2 vs. dm	1363	1774	3137
lr1 vs. er	21	4	25
lr2 vs. er	7	7	14
lr2 vs. lr1	4	8	12

Adjusted p-value threshold: 0.05. dm - development/maintenance, er – early regression, lr –late regression (lr1, lr2 – subgroups of lr)

**Fig. 3:**
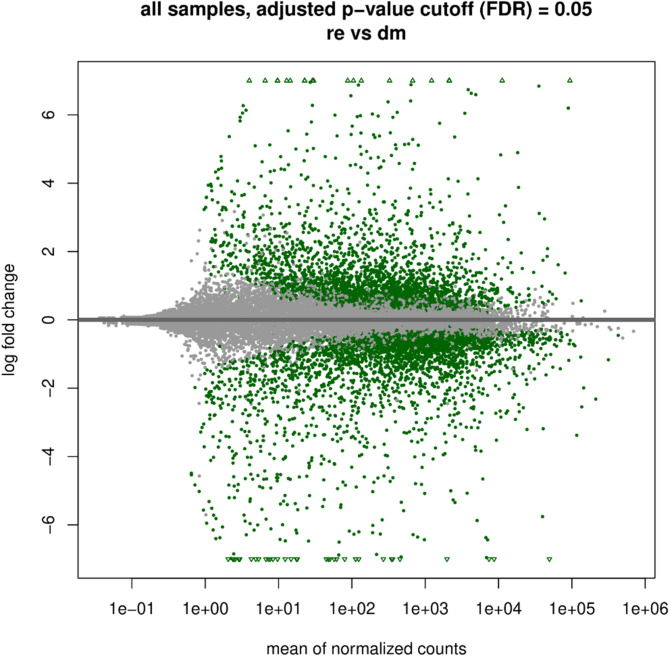
The MA-plot shows the log2-fold changes of each of the 21,701 genes over the mean of normalized counts for all the samples, applied on shrunken log2-fold changes, using the apeglm estimator. Points of genes are coloured if the adjusted p value is less than 0.05, tested against an LFC threshold of 0

In conclusion, we decided to combine all samples from regression stages (er, lr1, lr2) in the “regression” group. In all the results below, we continue to use these two main groups/stages: development/maintenance (dm) and regression (re). Differential gene expression (DGE) analysis revealed that 2882 genes were significantly upregulated, indicating that their expressions were higher in the re stage than in the dm stage, whereas 3292 genes were downregulated (supplemental file 3).The dispersion of the log fold changes is visualized in an MA plot (Fig. 3).

The top 50 down- and upregulated genes are listed in Table 3. The gene encoding proenkephalin (*PENK*) is the gene with the highest fold change in expression with respect to higher expression in dm compared to re, followed by the genes encoding claudin4 (*CLDN4*) and oxytocin prepeptide (*OXT*). In addition, genes encoding for progestin and AdipoQ receptor family member 5 (*PAQR5*) and corticotropin-releasing hormone binding protein (*CRHBP*) are listed among the 50 most downregulated genes. Interestingly, among the top 50 upregulated genes, genes encoding for three potential cholesterol- and/or steroid-metabolizing enzymes AKR1D1, CYP21A2 and CYP26A1, are listed and show drastically higher gene expression in re samples than in dm samples, all with fold change values of above 100. The protein expression of AKR1D1 was also investigated and a strongly different expression was confirmed (supplemental Fig. 1). The expression of selected genes (*AKR1D1*,* CYP21A2*,* and PAQR5*) was also analysed via quantitative real-time PCR to confirm the conformity of the gene expression data generated via RNA-Seq and quantitative real-time PCR (supplemental Fig. 2).

**Table 3:** Top down- and upregulated genes (comparison regression - re versus development/maintenance - dm). Here only genes are listed with have for the down-regulated genes values > 0 for all dm-samples and genes values > 0 for up-regulated genes in all re-samples

down = high in dm			up = high in re		
gene name abbreviation	full gene name	fold change	gene name abbreviation	full gene name	fold change
PENK	proenkephalin	-862.7	ENSFCAG00000024877	BRICK1 subunit of SCAR/WAVE actin nucleating complex	833.8
CLDN4	claudin 4	-606.0	FBXL16	F-box and leucine rich repeat protein 16	644.0
OXT	oxytocin/neurophysin I prepropeptide	-304.9	SERPINB3	serpin family B member 3	549.1
ENSFCAG00000030530	Elongation factor 1-alpha (UniProt match for transcript)	-300.9	CYP26A1	cytochrome P450 family 26 subfamily A member 1	392.2
ENSFCAG00000041846	“LncRNA, no protein”	-299.0	IL23R	interleukin 23 receptor	213.5
ARG1	arginase 1	-273.4	LOC101097497	keratin, type II cytoskeletal 5-like	196.9
AMTN	amelotin	-268.3	IL13	interleukin 13	184.7
APELA	apelin receptor early endogenous ligand	-267.0	CCL13	C-C motif chemokine ligand 13	179.3
SEC14L4	Section 14 like lipid binding 4	-263.6	CILP2	cartilage intermediate layer protein 2	165.5
PLA2G3	phospholipase A2 group III	-252.5	SERPIND1	serpin family D member 1	155.3
ENSFCAG00000049737	Cathepsin propeptide inhibitor domain-containing protein (UniProt match for transcript)	-225.7	PPP1R17	protein phosphatase 1 regulatory subunit 17	151.0
HSD3B1, HSD3B2	hydroxy-delta-5-steroid dehydrogenase, 3 beta- and steroid delta-isomerase 2	-169.1	PGLYRP2	peptidoglycan recognition protein 2	150.9
SLC5A1	solute carrier family 5 member 1	-162.5	CYP21A2	cytochrome P450 family 21 subfamily A member 2	145.2
ENSFCAG00000042967	“LncRNA, no protein”	-153.0	INSRR	insulin receptor related receptor	129.0
CNTN6	contactin 6	-140.6	AKR1D1	aldo-keto reductase family 1 member D1	117.9
FGA	fibrinogen alpha chain	-136.4	ENSFCAG00000040543	“LncRNA, no protein”	114.1
FABP6	fatty acid binding protein 6	-133.9	COL6A5	collagen type VI alpha 5 chain	112.5
RHCG	Rh family C glycoprotein	-133.6	PGAM2	phosphoglycerate mutase 2	112.1
LOC102902015	probable inactive serine protease 58	-133.3	SLC29A4	solute carrier family 29 member 4	102.1
PDC	phosducin	-131.3	TMEM213	transmembrane protein 213	101.9
GJE1	gap junction protein epsilon 1	-130.4	NAGS	N-acetylglutamate synthase	101.5
FGB	fibrinogen beta chain	-128.3	PTPRT	protein tyrosine phosphatase receptor type T	89.2
HSD17B3	hydroxysteroid 17-beta dehydrogenase 3	-126.7	ENSFCAG00000048400	“LncRNA, no protein”	77.8
DLK1	delta like non-canonical Notch ligand 1	-103.0	ENSFCAG00000051755	“LncRNA, no protein”	77.2
SERPINA3	serpin family A member 3	-102.4	TGFBI	transforming growth factor beta induced	75.8
IDO2	indoleamine 2,3-dioxygenase 2	-98.9	ATP6V0A4	ATPase H + transporting V0 subunit a4	75.5
FBP2	fructose-bisphosphatase 2	-96.2	LYPD6	LY6/PLAUR domain containing 6	73.3
ENSFCAG00000047393	“Uncharacterized protein”	-95.1	B3GALT2	beta-1,3-galactosyltransferase 2	73.0
ENSFCAG00000043251	“LncRNA, no protein”	-93.0	NTRK1	neurotrophic receptor tyrosine kinase 1	71.5
LHCGR	luteinizing hormone/choriogonadotropin receptor	-85.6	SLC4A8	solute carrier family 4 member 8	70.0
HAO2	hydroxyacid oxidase 2	-85.4	LOC101080976	pregnancy zone protein-like	68.6
PLPPR1	phospholipid phosphatase related 1	-84.1	TAS1R1	taste 1 receptor member 1	65.3
CPHL1	ceruloplasmin and hephaestin like 1	-83.6	MLXIPL	MLX interacting protein like	64.3
AREG	amphiregulin	-83.1	PERM1	PPARGC1 and ESRR induced regulator, muscle 1	56.8
UNC13C	unc-13 homolog C	-81.2	LEF1	lymphoid enhancer binding factor 1	55.4
ASIC2	acid sensing ion channel subunit 2	-81.2	CLEC4G	C-type lectin domain family 4 member G	49.8
SULT1C3	sulfotransferase family 1 C member 3	-79.2	RSPO4	R-spondin 4	48.7
SUCNR1	succinate receptor 1	-69.9	RHBG	Rh family B glycoprotein	48.5
“novel gene”	“LncRNA, no protein”	-69.4	ADIPOQ	adiponectin, C1Q and collagen domain containing	48.3
CPB1	carboxypeptidase B1	-63.7	BLK	BLK proto-onco, Src family tyrosine kinase	46.5
PAQR5	progestin and adipoQ receptor family member 5	-58.9	SLC6A4	solute carrier family 6 member 4	46.4
CRHBP	corticotropin releasing hormone binding protein	-58.2	FBXL21	F-box and leucine rich repeat protein 21	45.8
ADGRG7	adhesion G protein-coupled receptor G7	-54.5	INSYN2A	inhibitory synaptic factor 2 A	45.1
“novel gene”	“LncRNA, no protein”	-54.4	MME	membrane metalloendopeptidase	43.6
CPNE9	copine family member 9	-52.9	KCNK5	potassium two pore domain channel subfamily K member 5	40.8
ATP1A3	ATPase Na+/K + transporting subunit alpha 3	-51.6	CAPN13	calpain 13	40.2
TMPRSS7	transmembrane serine protease 7	-51.4	SVOPL	SVOP like	37.9
GAL3ST1	galactose-3-O-sulfotransferase 1	-49.7	TMEM221	transmembrane protein 221	37.1
“novel gene”	“LncRNA, no protein”	-49.5	NR4A2	nuclear receptor subfamily 4 group A member 2	36.5
SLCO5A1	solute carrier organic anion transporter family member 5A1	-49.3	ENSFCAG00000053105	“LncRNA, no protein”	36.4

**Fig. 4:**
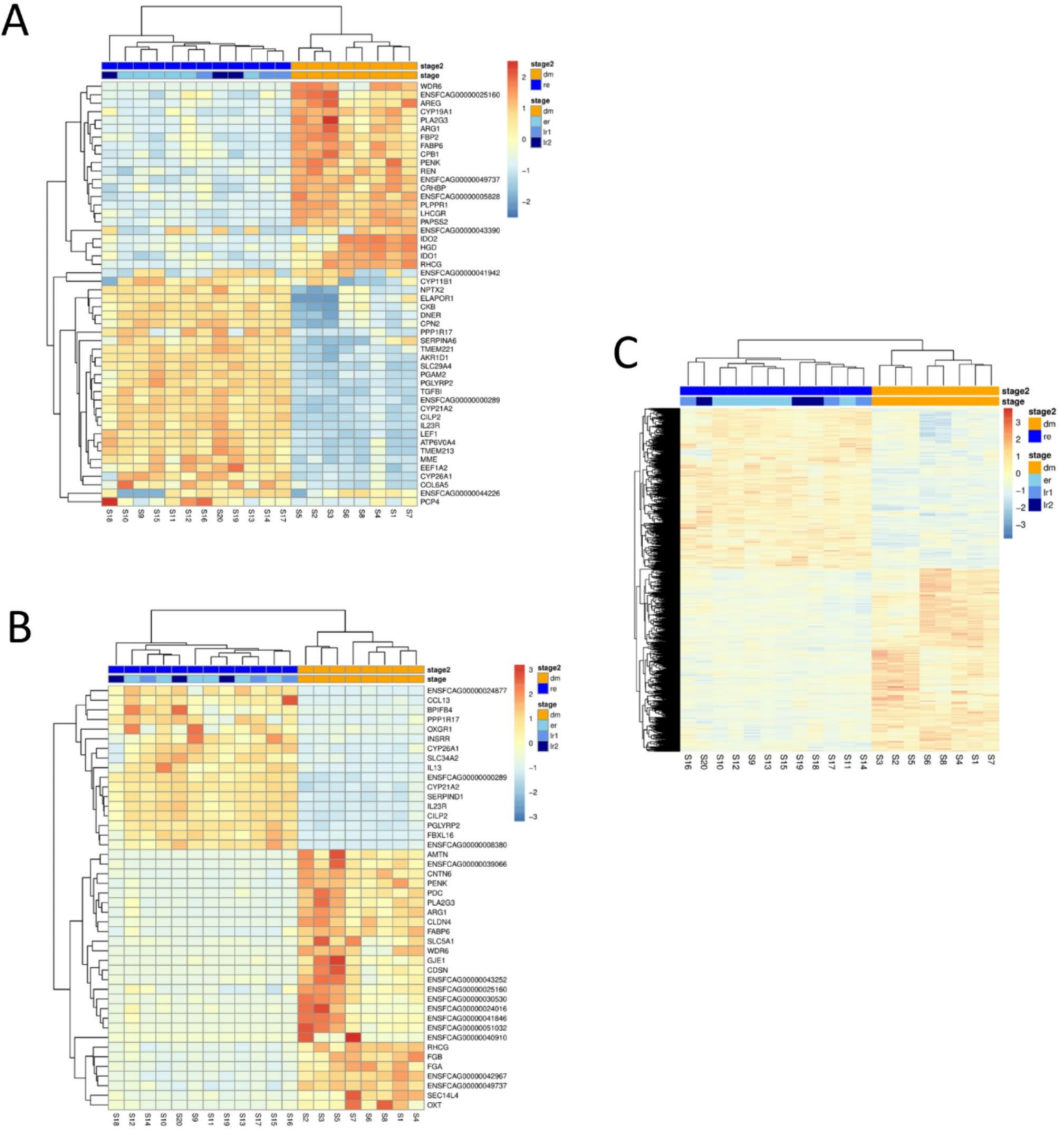
**A**: Heatmap of top 50 highly variable genes; **B**: Heatmap of DEGs with|log2FC| > 7; both visualized using z-scored values and hierarchical clustering of the genes. The Z-score gives the number of standard-deviations that a value is away from the mean of all the values in the same group, here the same gene. **C**: Heatmap of significantly differentiated genes (with adjusted p value $\:\le\:\:$0.05)

Furthermore, we have analysed the most variable genes, these are genes with the highest variance across samples independent of sample group relations. The top 50 genes are presented in a heatmap (Fig. 4A). The genes partly overlap with the genes on the top 50 differentially expressed genes list. As an additional heatmaps, we visualized DEGs with absolute log2-fold change values > 7 (Fig. 4B) and all DEGs (Fig. 4C). In neither heatmap, the substages er, lr1, and lr2 build single clusters.

### Functional analysis

We performed a functional annotation analysis to gain further insight into the physiological processes that differ between the two luteal stages.

In functional annotation cluster analysis using DAVID, 162 and 163 clusters with enrichment scores greater than 1 were identified for down- and upregulated genes, respectively (re versus dm comparison, supplemental file 5). The most prominent clusters of downregulated genes included, e.g., GO terms related to peptide synthesis (cluster 1), carboxylated acid processes (cluster 2), ribosomes and RNA (clusters 3–5, 9), mitochondria (clusters 6 and 11), lipid biosynthesis and metabolism (cluster 7), cholesterol and steroid biosynthesis and metabolism (cluster 12) and peroxisome (cluster 20). Clusters of upregulated genes contained GO terms related to zinc finger (cluster 1), transcription regulation (cluster 2), ion binding (cluster 4), negative regulation of biosynthesis and metabolic processes (cluster 5), lipid binding (cluster 7), positive regulation of signalling (cluster 10) and positive but also negative regulation of cell differentiation (clusters 11 and 14). Additionally, functional annotation charts are listed in the supplementary material (supplemental file 6). These terms included, e.g., the downregulated terms GO:0006695 ~ cholesterol biosynthetic process and GO:0006629 ~ lipid metabolic process and the upregulated terms GO:0006357 ~ regulation of transcription from RNA polymerase II promoter and GO:0031327 ~ negative regulation of cellular biosynthetic process.

### Pathway analysis

**Fig. 5:**
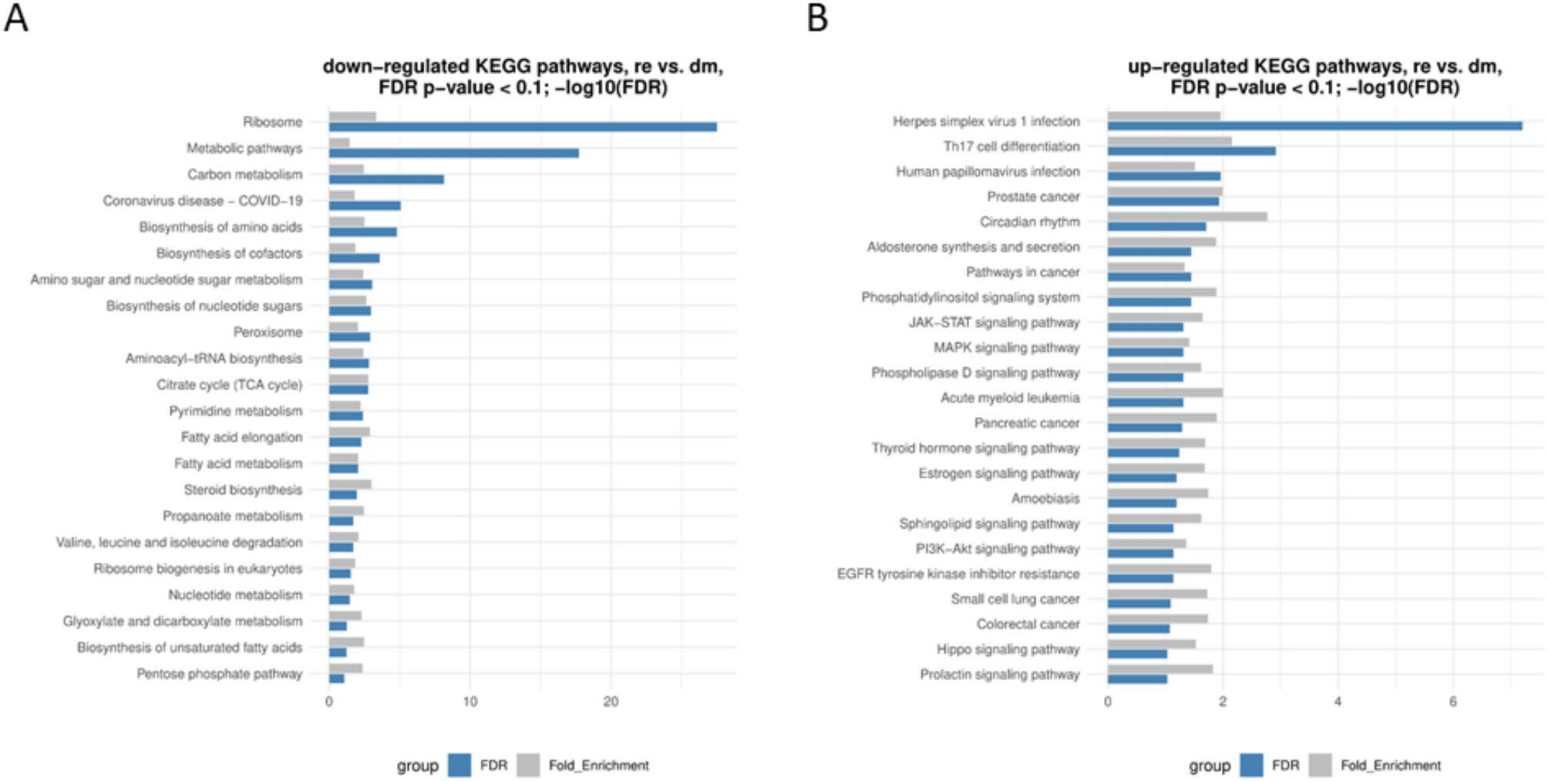
Bar graphs of KEGG pathways. **A**: down-regulated **B**: up-regulated. FDR = false discovery rate

The KEGG pathway analysis revealed the pathways shown in Fig. 5 and listed in supplemental file 7.

Stage dm (Fig. 5A – downregulated pathways) is characterized by an upregulation of genes encoding for ribosomal proteins and of pathways that are involved in the biosynthesis of diverse compounds, e.g., lipids, and metabolic pathways. In contrast, signalling pathways, cancer pathways, and infection pathways are dominating in the stage re (Fig. 5B). Selected pathways (e.g., steroid biosynthesis and aldosterone synthesis) are presented in more detail in supplemental file 8.

## Discussion

### Stages and pathways

This study characterizes, for the first time, the comparison of global gene expression in the CL during pseudopregnancy in domestic cats, with a focus on the development/maintenance (dm) and regression (re) stages. The very early stage of the life cycle, the formation stage, was excluded because not enough samples could be obtained. Furthermore, this stage is expected to be very heterogeneous due to the differentiation of progenitor cells into luteinized cells, which does not occur uniformly in time and space within the CL (our own observations). As the samples used in this study were obtained from animals for which the exact cycle day could not be determined, the classification of their stages was based on hormone content and histomorphological characteristics, as described in the Materials and Methods. Morphological and hormonal analyses allowed us to distinguish three different regression stages, but these could not be confirmed with the results of the RNA-Seq analysis, as a compact cluster was built in the PCA plot (Fig. 2A). Consequently, we decided to combine all the regression samples into one regression group. In contrast, samples of stage dm are widely spread in the PCA plot. This could correspond to different subphases in the development/maintenance stage. Cardoso et al. collected CLs from pseudopregnant dogs on days 10, 20, 30, 40, 50 and 60 post ovulation (po) and classified them as follows: 10 days po = developing, 20 days po = mature, 40 days po = early regression, and 60 days po = late regression. In the PCA plot, samples from the day 10, 20, 30 and 40 groups are closer to each other than to the other groups and samples. Samples from days 50 and 60 post ovulation are more dispersed and mixed and are clearly separated from the early regression of day 40 [18], which is in contrast to our results of samples from stage regression. We found one potential dm subgroup consisting of samples S2, S3 and S5, on the basis of their clustering both in the PCA plot and in all the heatmaps, which was just as separated in the PCA plot as the samples from day 10 po in the study of Cardoso et al. [18]. These dm samples may have been from the beginning of the development/maintenance phase and close to formation. Bharati et al. analysed the CLs of cycling pigs, which were classified as early, mid or late luteal and regression. They reported that luteal stages, which were developmentally closer, such as early versus mid and late versus regression, shared a greater number of common transcripts than early versus late or regression stages did [17]. This would mean that their late luteal stage would be more comparable to samples of our stage re. In Maiwa yak, the transcriptomes of CLs at early (EYCL), mid (MYCL) and late stages (LYCL) were studied [2], which correspond to estrus stages days 3–4, days 10–11 and days 15–16, respectively, as described previously [33]. The MYCL samples have a significantly higher P4 content than the EYCL and LYCL samples do [33], so the LYCL samples seem to have undergone functional regression.

As expected, we observed an enrichment of genes involved in sterol biosynthesis processes and cholesterol biosynthesis processes among the upregulated genes in dm. The same was observed by comparing stages in pigs, e.g., early luteal versus regressed, and mid luteal versus regressed luteal stages [17] and by comparing stages in dogs, e.g., 20 vs. 40 and 20 vs. 60 days po [18]. We identified numerous other pathways and GO terms in our study (see supplemental files 5–8), which are enriched either in stage dm or re, but, e.g., in contrast to Bharati [17], we could not observe an enrichment of “extracellular matrix organization”. There seem to also be other species-specific differences, e.g., in our study, the PI3K‒Akt pathway was upregulated in re. In contrast, it was the most enriched signalling pathway according to a comparison of stages early versus middle and middle versus late in Yak CL samples [2]. We suggest that functional pathways such as steroidogenesis are regulated rather similarly, as shown for cholesterol biosynthesis, whereas the regulation of the life cycle could proceed differently, as signals, e.g. for regression, are probably species specific. Future studies should analyse whether there are differences in the transcriptomes of CLs from pseudopregnancy and pregnancy in domestic cats at the same morphologically assessed stage, as well as between cyclic corpora lutea and those with a prolonged lifespan due to pregnancy in other species. We hypothesize that there are likely differences that support the prolonged life span of CL during pregnancy.

### Potential ways for cholesterol efflux and metabolism of cholesterol and steroids during the regression stage of CLs

The potential cholesterol- and/or steroid-metabolizing enzymes AKR1D1, CYP21A2 and CYP26A1 are of particular interest in our study, as their gene expression is remarkable higher in re stage than in dm stage (fold changes: *AKR1D1* = 118, *CYP21A2* = 145, *CYP26A1* = 393).

AKR1D1 is a member of the aldoketo reductase superfamily and functions as a Δ4-3-ketosteroid-5β-reductase. It generates 5β-dihydrosteroids [34] and is well known to be involved in bile acid biosynthesis, which also serves as a major pathway for cholesterol metabolism [35]. It is therefore possible that the potential excess cholesterol in the regressing CL is metabolized in this way. Alternatively, this enzyme could also metabolize progesterone or other sex steroids in regressing CLs, as this enzyme was also shown to interact with these steroids [36, 37]. Interestingly, 5β-steroids can be active metabolites, e.g., 5β-pregnanes are neurosteroids devoid of progestogenic effects and seem to suppress myometrial contraction (see review [34]). *AKR1D1* is part of annotation cluster 62 “up” in supplemental file 5, GO terms connected with it are sterol, steroid and cholesterol catabolic processes. It is not yet known which metabolites formation is supported by AKR1D1 in cat CLs and which function the metabolites could have for CLs of regression or for other organs/tissues.

*CYP21A2* is listed in three annotation clusters in supplemental file 5 “up” and part of GO terms regarding ion, lipid or cyclic compound binding. Its gene product is an enzyme of the biosynthesis pathway of glucocorticoids and mineralocorticoids, which catalyze the 21-hydroxylation of progesterone and 17OH-progesterone, respectively [38]. Strong expression of this enzyme could therefore lead to fast conversion of progestogens to steroids that are not sex steroids, reducing the sex steroid level in CLs. If the potential synthesized glucocorticoids and/or mineralocorticoids are indeed increasing and have an intraluteal or extraluteal function, could be examined in the future in more detail. The genes encoding the glucocorticoid receptor (NR3C1) and mineralocorticoid receptor (NR3C2) are also expressed differently, with significantly higher expression in stage re. Interestingly, the profile of *CYP21A2* expression in domestic cat CLs is different from that of *CYP21A2* in bovine CLs. There, the *CYP21A2* expression in the four analysed CL stages followed the production profile of P4, with lower levels in the early and late stages [39]. In humans, *CYP21A2* expression in luteinized granulosa cells (LGCs) is correlated with the lipid content in these cells, and it was concluded that they likely synthesize 21-hydroxylase-derived mineralocorticoids from cholesterol-containing lipids in vivo to promote postovulatory luteinization via mineralocorticoid receptor-mediated events [40]. Owing to the very high gene expression in stage re in domestic cats, we suppose a connection to regression processes of this enzyme, e.g., by P4-inactivation.

CYP26A1 (cytochrome P450 family 26 subfamily A member 1) is an enzyme known for its role in retinoic acid (RA) metabolism and homeostasis in mammals [41]. Its gene is listed in the same clusters as *CYP21A1* and in another one of supplemental file 5 “up”; here connected with the GO term “GO:0070887 ~ cellular response to chemical stimulus”. However, a cytochrome P-450 enzyme that catalyses the 26-hydroxylation of C27 steroids has also been described previously [42], and Yohida et al. reported that CYP26A1 is an enzyme that catalyzes among others the 26-hydroxylation of cholesterol [43]. The conversion of cholesterol to 26-hydroxycholesterol seems to block cholesterol utilization in the cell. This group described this enzyme as a negative regulator of progesterone in the rat CL and reported an increase in its gene expression as pseudopregnancy progressed [43]. This finding aligns with our observation of high gene expression in regression samples. We also cannot exclude an effect of RA and its metabolites in the domestic cat CL on the life cycle and functional regulation via their potentially different ratios and/or interactions with their receptors. In the dog CL, the gene expression of components of the liver X receptor-retinoid X receptor (LXR-RXR) signalling pathway was studied in more detail. The cholesterol availability regulated by this pathway seems to be locally tuned and could contribute to luteal regression of the canine pseudopregnant CL [18]. For some of the LXR-related genes listed previously [18], we detected differences in gene expression between stages dm and re. *CYP51A1*, *HMCGR*, *IGFBP3*, *IGFBP4*, *IL1R2*, *LDLR*, *NR1H3*, *PTGS2*, *SCARB1* and *TNFRSF1A* are higher expressed in dm; *ABCA1*, *ABCG1*, *CYP27A1*, *IL1R1*, *NCOR1*, *NCOR2* and *NR1H2* are higher expressed in re in our study. The LXR-RXR signalling pathway could influence “reverse cholesterol transport” [18], which is the efflux of excess cellular cholesterol from peripheral tissues and its return to the liver for excretion in the bile and ultimately the faeces. The transporter proteins ABCA1 and ABCG1 can work together to promote this process [44]. *ABCA1* and *ABCG1* were higher expressed in re samples than in the dm samples in our study and could therefore support cholesterol efflux especially at the stage re. Interestingly, the genes encoding the liver X receptor variants LXRA and LXRB (*NR1H3* and *NR1H2*) were regulated in opposite directions in our study; *NR1H3* was higher expressed in stage dm, and *NR1H2* was higher expressed in stage re. In the canine CL, no significant differences in the gene expression of *NR1H3*, *NR1H2* or *ABCG1* were observed, but *ABCA1 was* upregulated on day 50 p.o [18]. Similarly to our study, the gene expression of *ABCA1* and *ABCG1* was higher and the expression of the lipoprotein receptors *LDLR* and *SCARB1* was lower in the functionally regressed CL of macaques than in the functional mid-late CL [45]. This group suggested that a possible mechanism underlying the loss of P4 production in the primate CL may involve a restriction of cholesterol availability through inhibited lipoprotein uptake (LDLR, SCARB1), decreased de novo synthesis of cholesterol (HMGCR, SQLE), and increased cholesterol efflux from the CL (ABCA1, ABCG1). For ovine cyclic CLs, it was suggested that the reduction in lipoprotein receptors rather than LXR-mediated reverse transport might contribute to the decline in progesterone (P4) during natural and functional luteolysis [46].

On the basis of our results, we propose that in addition to a decrease in lipoprotein receptor expression, an increase in cholesterol efflux, and a reduced steroidogenic activity, increased progesterone and cholesterol metabolism could play a role in P4 reduction at the regression stage of domestic cat CLs.

### Gene expression of progesterone receptors

We detected transcripts for the classical nuclear progesterone receptor (*PGR*), for progesterone receptor membrane compounds 1 and 2 (*PGRMC1 and PGRMC2*) and for progesterone membrane receptors (*PAQR3*,* PAQR4*,* PAQR5*,* PAQR6*,* PAQR7*,* PAQR8*,* and PAQR9*) in our samples, but only two of them were differentially *expressed—PAQR5* and *PGRMC1*. Most interesting is the large fold change for PAQR5 (also known as mPRγ), with a 59-fold higher gene expression in stage dm than in stage re. In terms of the expression levels of *PGR*, *PAQR7*, *PAQR8*, *PAQR5*, and *PGRMC1* in total bovine CL tissue on day 11 versus day 18, *PAQR7* and *PAQR8* increased, *PGR* and *PGRMC1* decreased, whereas *PAQR5* expression remained constant [47]. The gene expression levels of mPRα, mPRβ and mPRγ (= PAQR7, PAQR8 and PAQR5) increased in the bovine CL during the oestrus cycle but increased only in mPRγ in the CL of pregnancy [48]. In contrast to the increase in the gene expression levels of PAQR genes in samples from later luteal phases in bovine CLs, in our study, the expression level of *PAQR5/mPRγ* was higher in stage dm and lower in stage re, and the difference in expression was restricted to this membrane receptor only. In another study, the gene and in part also the protein expression of selected progesterone receptors (PAQR5, PAQR7, PAQR8, PGRMC1, PGRMC2 and PGR) in the CL of bitches with different reproductive statuses was investigated; all the named receptors were expressed in all the tested statuses [49]. In a study of CLs of yaks, *PRGMC1* was more highly expressed in middle-stage CLs than in early- or late-stage CLs [2]. This finding aligns with our observation of higher expression in dm than in re. Yang et al. further showed that the inhibition of *PGRMC1* expression in luteinized granulosa cells by RNAi resulted in a reduction in P4 in the supernatant of the cells [2]. Comparisons of our data with data from other species suggest that in all these species, different types of progesterone receptors are present in the CL so that progesterone can act through genomic and nongenomic mechanisms, but there are obviously species-specific expression patterns that could lead to different regulatory effects.

### CRH/UCN-receptor-binding protein (UCN-R-BP) system

*CRHBP* (coding for corticotropin-releasing hormone binding protein) is a highly variable gene in domestic cat CLs (see Fig. 4A) and has a fold change value of 58, which corresponds to a 58-fold higher gene expression in stage dm than in stage re. We could not find its gene name listed in one of the clusters of supplemental file 5. Knowledge about the function of this protein in the CL is limited. Corticotropin-releasing hormone (CRH) is one of the principal modulators of the hypothalamic–pituitary–adrenal (HPA) axis but is not exclusively produced in the hypothalamus [50]. During human pregnancy, the plasma level of circulating maternal immunoreactive CRH increases strongly from the first trimester of gestation due to the CRH production in the placenta [51]. Suda et al. reported that CRH found in pregnant women can bind to CRHBP and thus is inactivated. Therefore, plasma ACTH levels may not increase to above the normal range during pregnancy [52]. Interestingly, other genes encoding for CRH/UCN-receptor-binding protein (UCN-R-BP) system members, including CRH, UCN, UCN2, UCN3, CRHR1, and CRHR2 [53], were not expressed or were nearly not expressed in our sample set. This is in contrast to macaque CL, where gene expressions of CRH, UCN, UCN2, CRHR1, CRHR2 and CHRHBP were detected [54]. *CHRBP* expression is highest in late and very late CLs of the natural menstrual cycle [54], which is in strong contrast to our study, which revealed higher expression in stage dm than in stage re. Therefore, we can only speculate that, in domestic cats, the potential protective and/or regulatory function of CRHBP provided by the CL is greater in times of active CLs than of regressed CLs. However, on the basis of the very low gene expression of the CRH receptors CRHR1 and CRHR2, a direct function of CRH in domestic cat CLs seems unlikely. CRHBP expression in CLs potentially protects the surrounding ovarian tissue or the whole ovary if it is not even released into the bloodstream. CRH can suppress ovarian steroidogenesis in vitro, e.g., in cultured human granulosa-lutein cells [55] or thecal cells of human ovarian follicles [56]. The potential protective role of CRHBP should be analysed in more detail in the future.

### Adipokines and their receptors

Adipose tissue synthesizes and secretes peptides/proteins called adipokines. They play a critical role in the development of obesity-related complications and inflammatory conditions. However, they are also involved in other functions in organisms, including reproductive functions [57]. In our study, we could not detect transcripts for the adiponectine vaspin (visceral adipose-specific serpin, *SERPINA12*). Gene for Chemerin (retinoic acid receptor responder 2, *RARRES2*) is highly expressed in the domestic cat CL, but there are no differences between the two stages, whereas gene for visfatin (nicotinamide phosphoribosyltransferase, *NAMPT*) is more highly expressed in stage dm than in stage re (fold change = -1.97). The strongest difference in gene expression was observed for adiponectin, also called adiponectin, C1Q and collagen domain-containing (*ADIPOQ*, fold change = 48), with nearly no expression in dm samples. *ADIPOQ* is part of many clusters and a diversity of GO terms (see supplemental file 5, “up”). In contrast to *ADIPOQ*, the adiponectin receptor 2 (*ADIPOR2*) gene was more highly expressed in the stage dm than in the stage re (fold change = -2.4). The gene expression of its second receptor, ADIPOR1, was not detectable. A study on bovine CLs revealed that growing CLs have lower gene expression than regressed CLs for adiponectin, ADIPOR1 and ADIPOR2 [58]. In buffalo, adiponectin, ADIPOR1, and ADIPOR2 are present in CL throughout the oestrous cycle, but their expression levels are higher in the early and regression stages and lower in the middle and late stages of the CL [59]. Our results of higher expression levels of *ADIPOQ* and lower expression levels of *ADIPOR2* in regression CLs differ partly from these studies. It remains unclear which specific function adiponectin and its receptors have in CLs. Anuradha et al. hypothesized that the high concentration of adiponectin in the CL could prevent apoptosis in luteal cells despite a decline in progesterone synthesis during the period of delayed embryonic development of *Cynopterus sphinx* [60]. Beside different reproductive effects (see review of [61]), adiponectin is known for its pleiotropic effects on the coordination of adipose tissue expansion and vascularization, anti-inflammatory effects, increased metabolic flexibility, improved insulin sensitivity, improved skeletal muscle function, cardiovascular function, and liver function [62].

Although the gene expression of apelin (APLN) and apelin receptor (APLNR/APJ) was very low in our sample set, we detected transcripts of the Apelin Receptor Early Endogenous Ligand (*APELA*,* ELA*,* ELABELA*), which is another ligand for the apelin receptor. *APELA* was highly differentially expressed (fold change = -267) (see Fig. 4B as ENSFCAG00000039066). This gene is expressed mainly in stage dm, and no or only a few transcripts were detected in the regression samples. To our knowledge, there are studies on APELA in the female reproductive tract (see review to APLN and APELA of [63]), but CLs have not been analysed. APELA is detected in the placentas of both mice and humans and is discussed as a circulating hormone that ensures the cardiovascular integrity of both mothers and fetuses during pregnancy. Its deficiency promotes preeclampsia and cardiovascular malformations in mice [64]. It is possible that in domestic cats, CLs are an additional or the only source of this hormone to fulfil the suggested functions during pregnancy. However, as we analysed the CL of nonpregnant cycles in the present study, there could also be another function for the APELA of this source. An intraluteal function seems to be unlikely due to the low gene expression of its receptor APLNR.

Leptin is known mainly for its function in energy metabolism, but it also seems to have reproductive functions (see review of Childs et al. [65]. On the basis of our gene expression results, leptin does not seem to be produced in the domestic cat CL. This seems to contrast with the CL of many other species, such as human rats, pigs, cattle, horses, goats and water buffaloes (see review [66]). Mlyczynska et al. assumed that leptin affects not only steroid hormone secretion but steroids can also affect leptin expression [66]. For example, in explants of horses of early and mid CL, a low leptin concentration had a stimulatory effect on P4 production, which was neutralized by ghrelin addition; ghrelin alone decreased P4 production [67]. Slightly different, in the bovine CL, an effect of leptin on luteal progesterone production in vitro was also detected, but leptin alone was ineffective and had to be combined with IGF-1 to stimulate P4 production [68]. Reversely, the gene expression of leptin in porcine luteal cells can be stimulated by LH, E2 and P4 [69]. In our study, we detected significantly higher gene expression of *LEPR* (which encodes for the leptin receptor) in regression CLs than in dm CLs (fold change = 15). This indicates that less active CLs are likely more sensitive to leptin than active CLs are; therefore, a positive effect of leptin on steroidogenesis seems unlikely in cats. In contrast to our study, the expression of LEPR (gene and protein) was higher in the early and mid CLs than in the late CLs of horses [67]. In canine CLs, leptin and leptin receptor expression was studied in CLs from pregnant and nonpregnant bitches. Transcripts for both genes were detected. In CLs of pregnancy, no differences in the expression of *LEP* or *LEPR* were detected over the studied period, nor were differences in the expression of *LEPR* in nonpregnant CLs. *LEP* expression was highest on day 35 p.o. in nonpregnant CLs [70]. Our results are remarkably different from the patterns observed in other species. The specific functions of leptin and its receptors in domestic cats need to be studied in more detail in the future. This example supports the hypothesis that some regulatory processes of CL function and life cycle could differ strongly between species.

### Limitations of the study

Our samples are from routine castrations, i.e. we know almost nothing about the previous history of the animals, nor do we know how many days before the ovariectomy the ovulation took place that led to the development of the CLs examined. We therefore had to classify them on the basis of morphological and endocrinological characteristics only and a direct comparison to other CL studies is limited.

We observed moderate mapping rates of reads to the *Felis catus* transcriptome. Usually, mapping rates from 70 to 90% are expected when mapping reads against human genome [71]. Here, we mapped against the transcriptome, thus lower mapping rates were expected. Further, a limited *Felis catus* annotation of the transcriptome could be responsible for the lower mapping rates. Besides, also preparation and mRNA isolation from samples can always influence mapping rates. Nonetheless, sufficient coverage of the transcriptome of *Felis catus* was reached enabling the identification of differently expressed genes and corresponding pathways. In total we identified 21,701 genes out of 29,550 genes.

## Conclusion

Transcriptomic analysis of domestic cat CL at the dm and re stages revealed a notable number of DEGs between these stages. Interestingly, we identified different possible ways of cholesterol removal in regression. Furthermore, we detected differential gene expression for potentially new regulatory factors. They are either hormones and may be secreted by the CL, e.g., some adipokines, a hormone-interacting protein, which could reduce the effect of the ligand by binding it (CHRBP) or hormone receptors that could transmit a hormonal effect intraluteal (e.g., LEPR and PAQR5). These findings indicate that the endocrine function of CLs is not restricted to the secretion of progesterone. For the expression of some genes studied in more detail, we observed different profiles than those reported in studies in other species. This finding supports the hypothesis that corpora luteum life cycles are partly differentially regulated between species.

Ethic statement.

This study was approved by the Internal Committee for Ethics and Animal Welfare of the IZW (2017-02-02).

## Electronic supplementary material

Below is the link to the electronic supplementary material.

Supplementary Material 1

Supplementary Material 2

Supplementary Material 3

Supplementary Material 4

Supplementary Material 5

Supplementary Material 6

Supplementary Material 7

Supplementary Material 8

Supplementary Material 9

Supplementary Material 10

## Data Availability

Bulk RNA-Seq raw data are deposited in European Nucleotide Archive (ENA) under Accession no: PRJEB82722.
