# Supplementary figures and images for: Transcriptome analysis of corpora lutea in domestic cats (Felis catus) reveals strong differences in gene expression of various hormones, hormone receptors and regulators across different developmental stages
Source: BMC Genomics. 2025 Mar 31;26:325. doi: 10.1186/s12864-025-11510-3 (PMC11959938; doi:10.1186/s12864-025-11510-3)

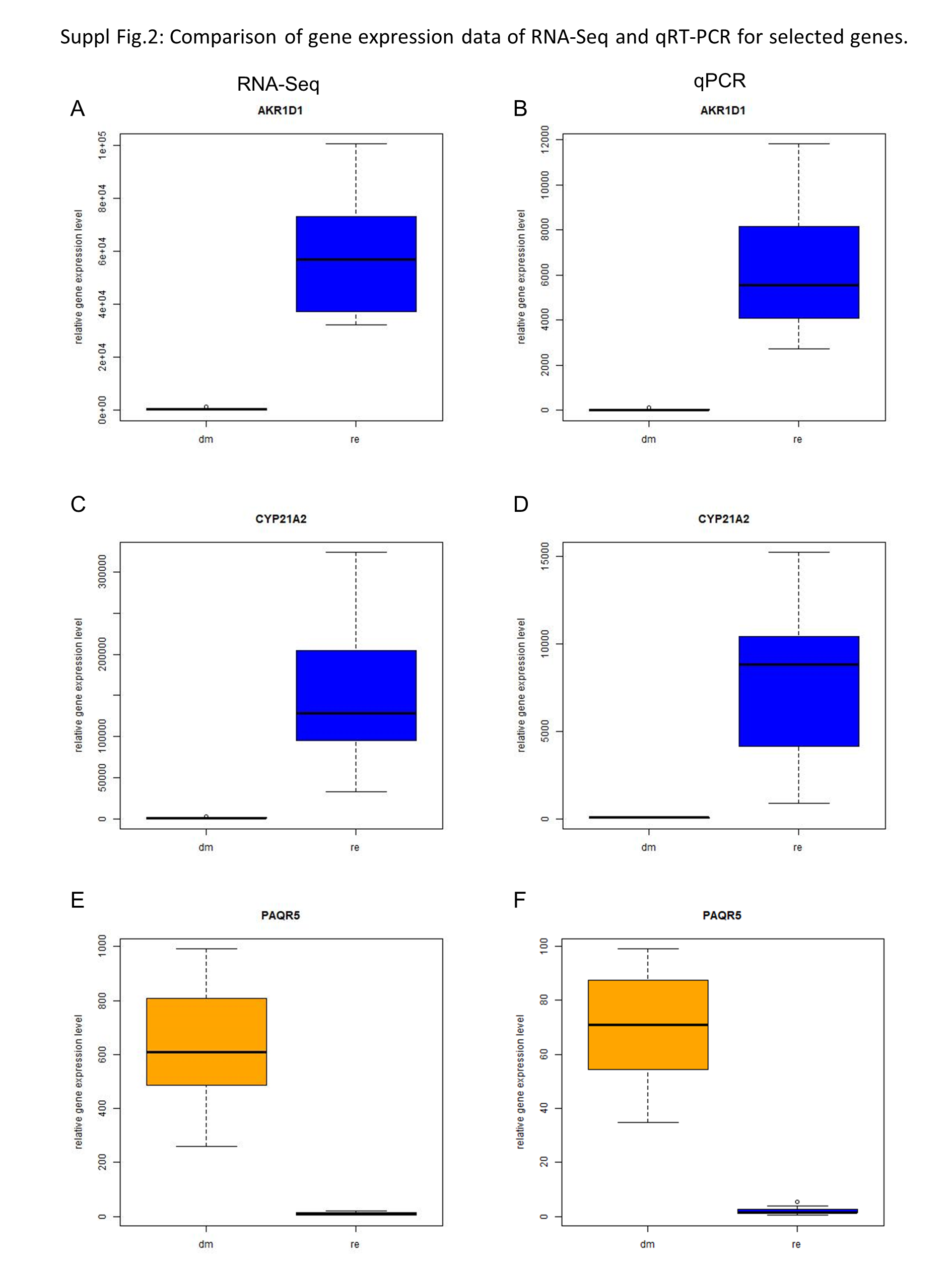

Supplement: Supplementary file 1 — Supplementary Material 1 [file 12864_2025_11510_MOESM1_ESM.tif]
