# Supplementary material for: Transcriptome analysis of corpora lutea in domestic cats (Felis catus) reveals strong differences in gene expression of various hormones, hormone receptors and regulators across different developmental stages
Source: BMC Genomics. 2025 Mar 31;26:325. doi: 10.1186/s12864-025-11510-3 (PMC11959938; doi:10.1186/s12864-025-11510-3)

Suppl Fig. 1.: Western Blot against AKR1D1 and beta Actin (control)

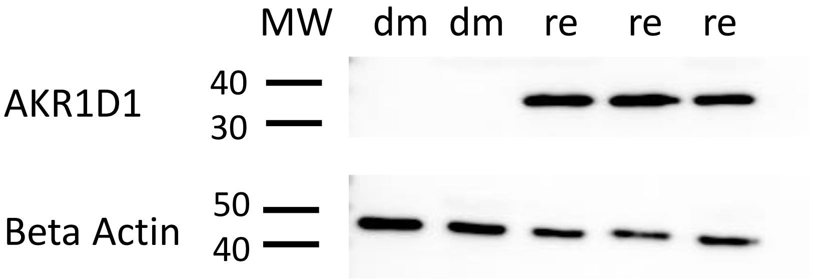

dm – development/maintenance, re – regression
MW – molecular weight. description of MW in kDa
AKR1D1 – aldo-keto reductase family 1 member D1

Original blot, lane description was added:

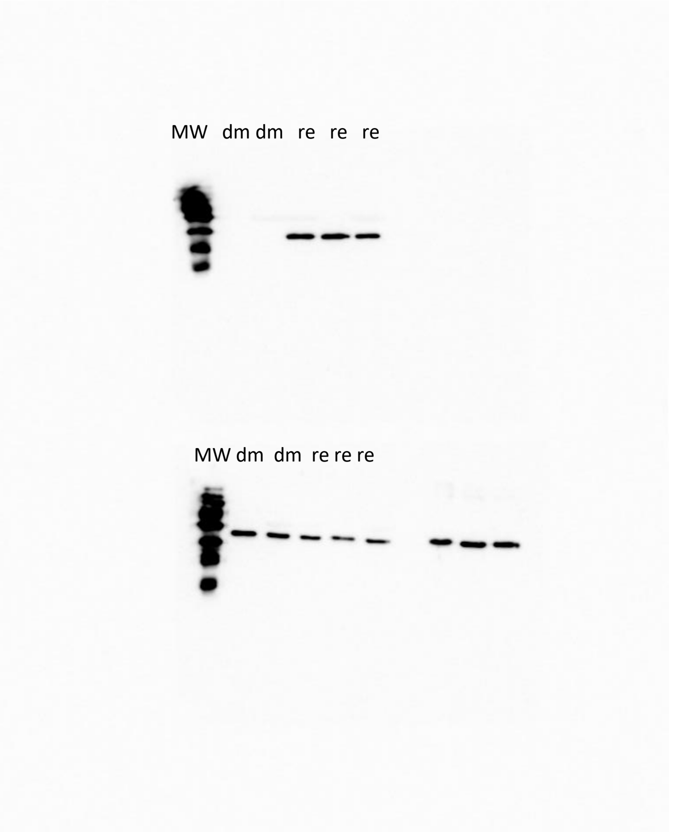

Autoimage of the Imager; combines chemiluminescence signals with a image of the membranes

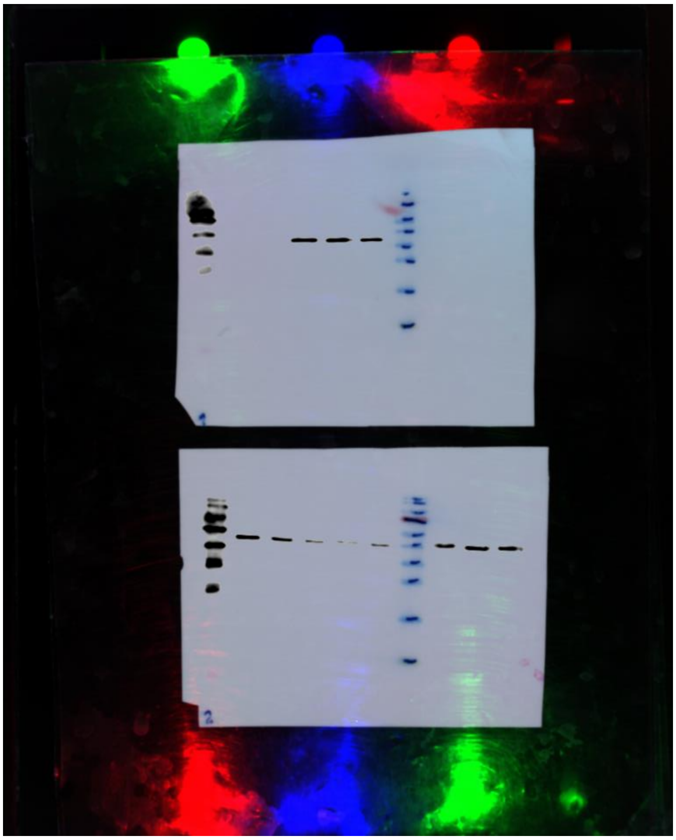

Supplement: Supplementary file 2 — Supplementary Material 2 [file 12864_2025_11510_MOESM2_ESM.pdf]
