# Supplementary material for: Transcriptome analysis of corpora lutea in domestic cats (Felis catus) reveals strong differences in gene expression of various hormones, hormone receptors and regulators across different developmental stages
Source: BMC Genomics. 2025 Mar 31;26:325. doi: 10.1186/s12864-025-11510-3 (PMC11959938; doi:10.1186/s12864-025-11510-3)
Supplement: Supplementary file 7 — Supplementary Material 7 [file 12864_2025_11510_MOESM7_ESM.pdf]

examples of regulated pathways

The color key gives the range (log2FC: -1 to 1), values beyond that range are converted to the closest extreme, i.e. log2 fold change (lfc) values > 1 are converted to the value 1, and log2 fold change (lfc) values < -1 are converted to the value -1.

examples of downregulated pathways

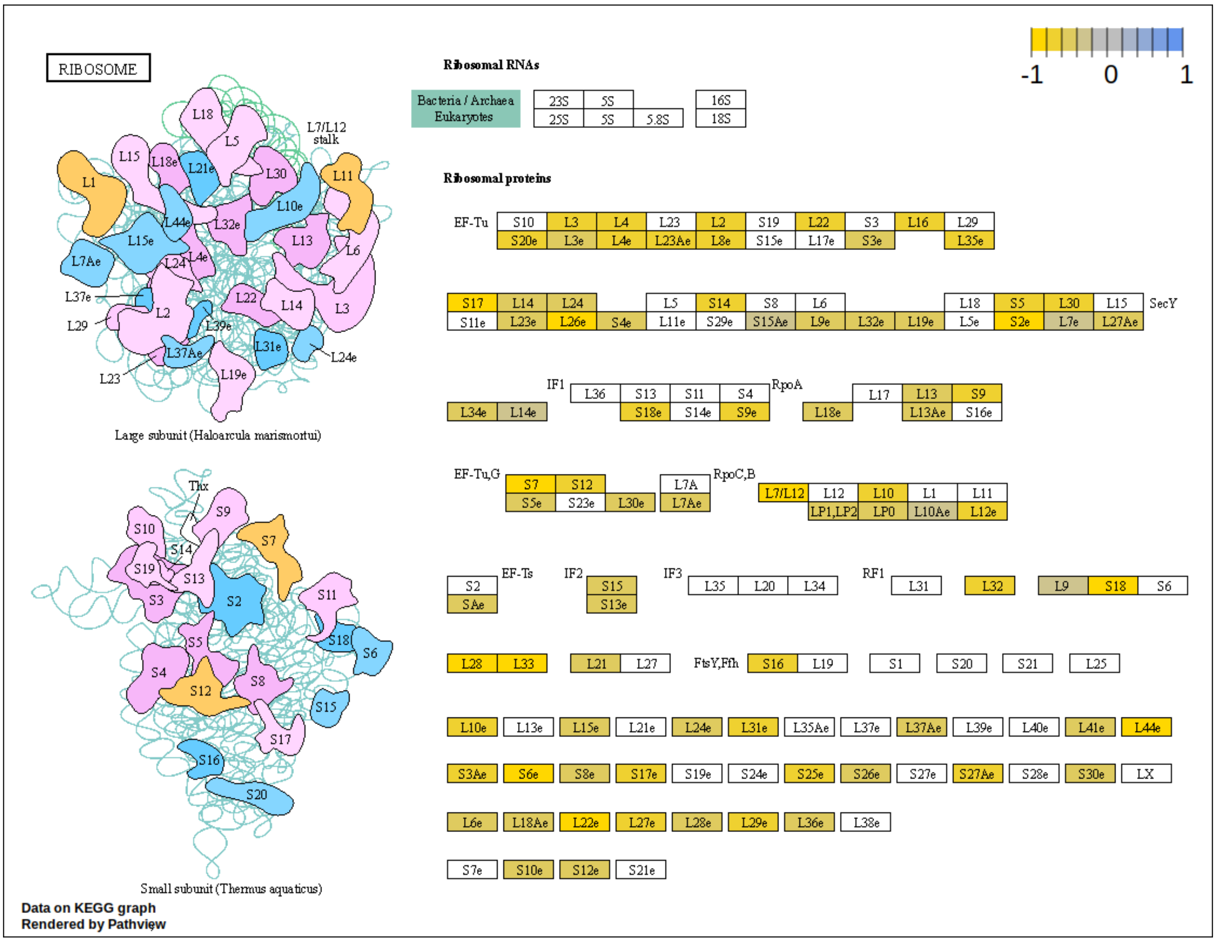

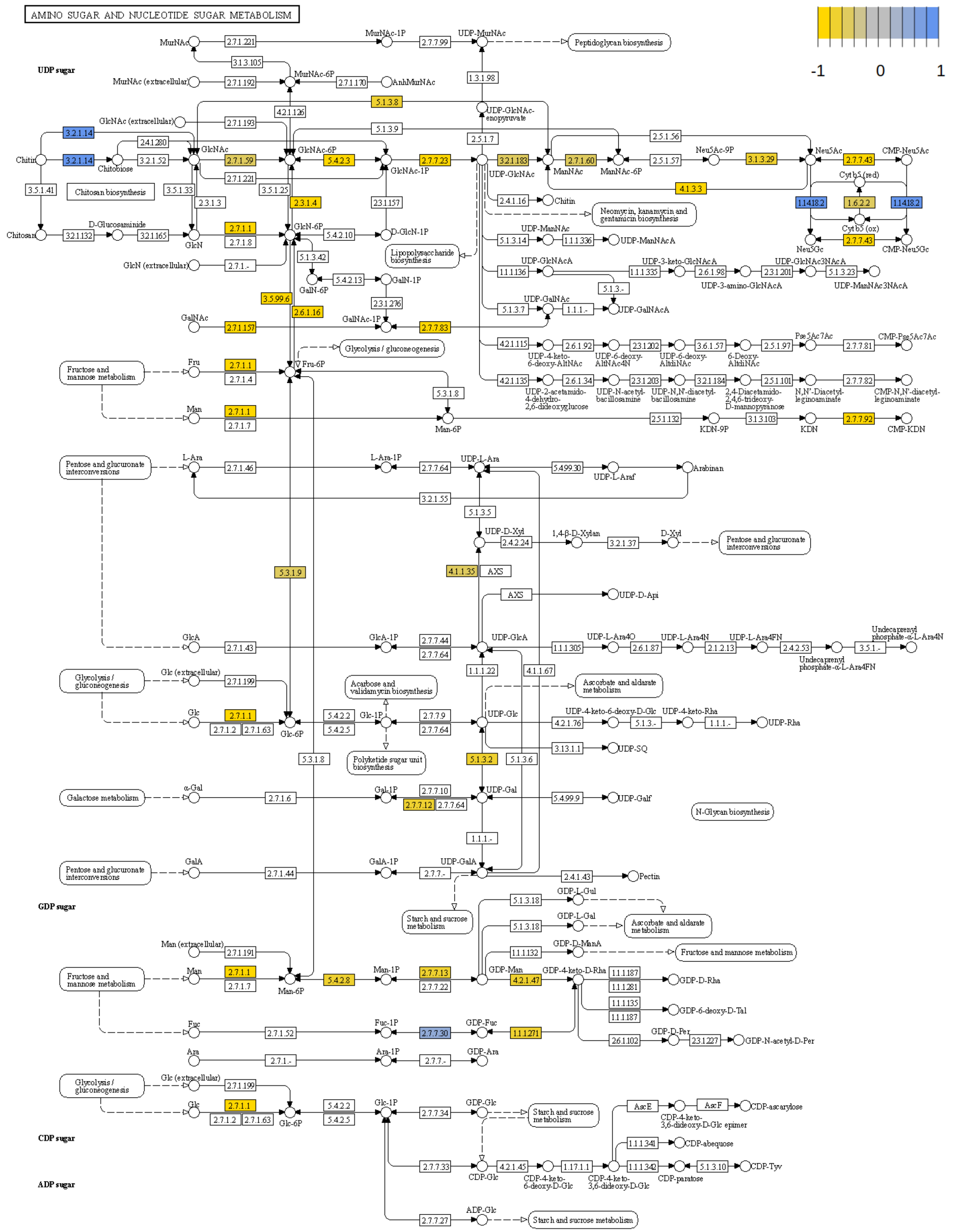

Data on KEGG graph
Rendered by Pathview

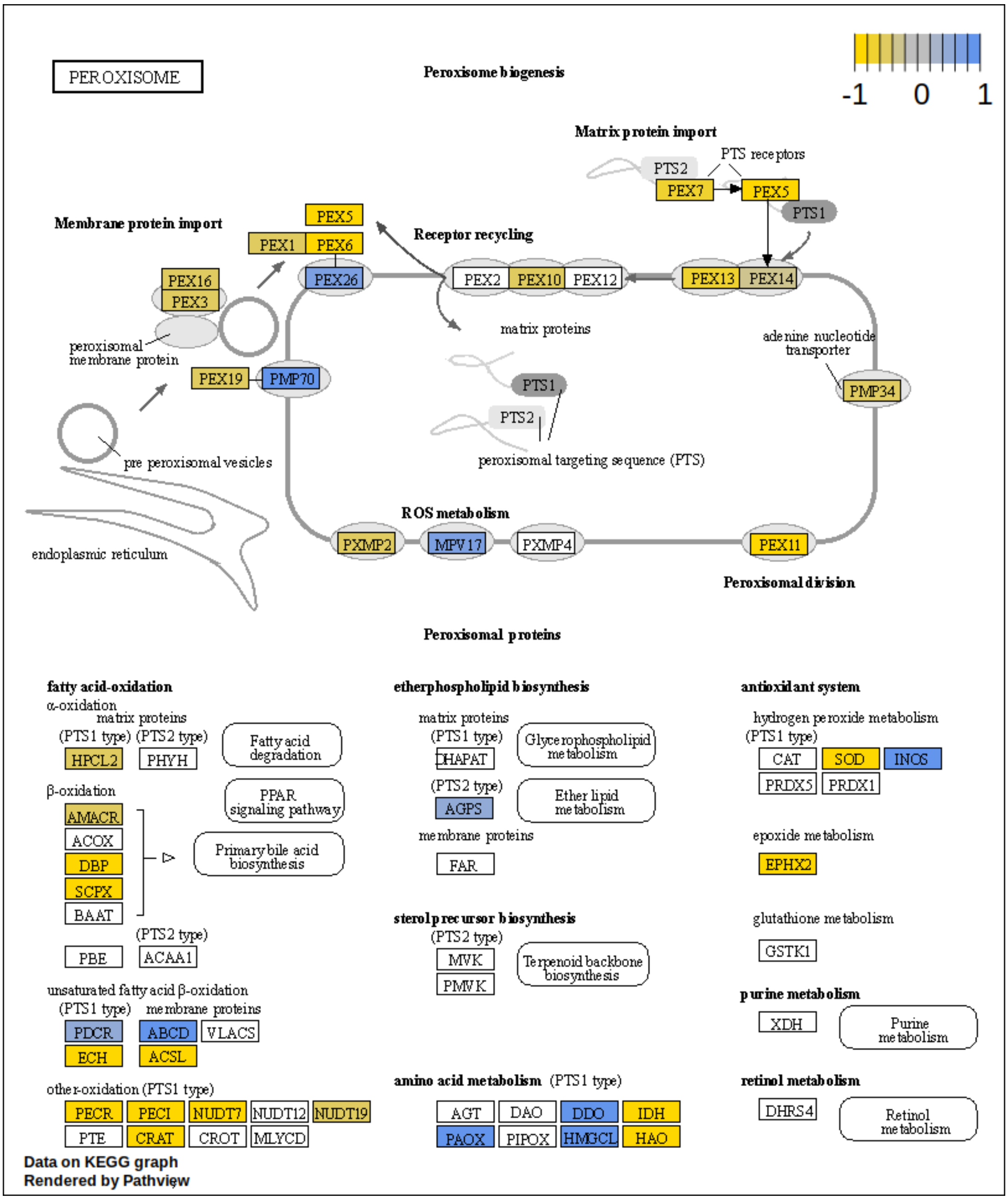

PEROXISOME
Peroxisome biogenesis
-1 0 1
Matrix protein import
PTS receptors
PTS2
PEX7
PEX5
PTS1
Membrane protein import
PEX5
PEX1
PEX6
Receptor recycling
PEX16
PEX3
PEX26
PEX2
PEX10
PEX12
PEX13
PEX14
peroxisomal membrane protein
matrix proteins
adenine nucleotide transporter
PEX19
PMP70
PTS1
PMP34
PTS2
pre peroxisomal vesicles
peroxisomal targeting sequence (PTS)
endoplasmic reticulum
ROS metabolism
PXMP2
MPV17
PXMP4
PEX11
Peroxisomal division
Peroxisomal proteins
fatty acid-oxidation
α-oxidation
matrix proteins
(PTS1 type) (PTS2 type)
HPCL2
PHYH
Fatty acid degradation
β-oxidation
PPAR signaling pathway
AMACR
ACOX
DBP
SCPX
BAAT
Primary bile acid biosynthesis
(PTS2 type)
PBE
ACAA1
unsaturated fatty acid β-oxidation
(PTS1 type) membrane proteins
PDCR
ABCD
VLACS
ECH
ACSL
other-oxidation (PTS1 type)
PECR
PECI
NUDT7
NUDT12
NUDT19
PTE
CRAT
CROT
MLYCD
etherphospholipid biosynthesis
matrix proteins
(PTS1 type)
DHAPAT
Glycerophospholipid metabolism
(PTS2 type)
AGPS
Ether lipid metabolism
membrane proteins
FAR
sterol precursor biosynthesis
(PTS2 type)
MVK
PMVK
Terpenoid backbone biosynthesis
amino acid metabolism (PTS1 type)
AGT
DAO
DDO
IDH
PAOX
PIPOX
HMGCL
HAO
antioxidant system
hydrogen peroxide metabolism
(PTS1 type)
CAT
SOD
INOS
PRDX5
PRDX1
epoxide metabolism
EPHX2
glutathione metabolism
GSTK1
purine metabolism
XDH
Purine metabolism
retinol metabolism
DHRS4
Retinol metabolism
Data on KEGG graph
Rendered by Pathview

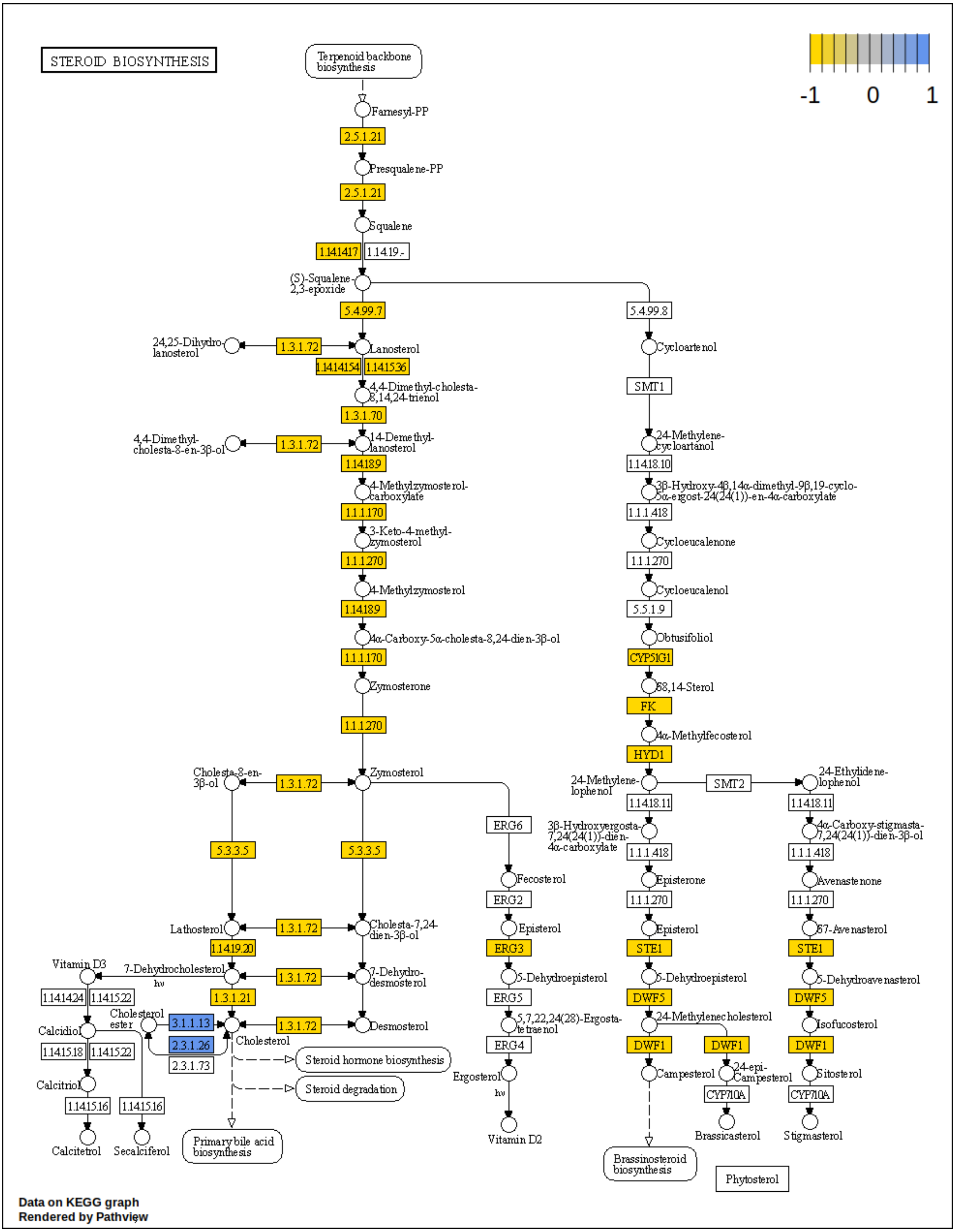
STEROID BIOSYNTHESIS
Terpenoid backbone biosynthesis
-1
0
1
Farnesyl-PP
2.5.1.21
Presqualene-PP
2.5.1.21
Squalene
1.14.14.17
1.14.19.-
(S)-Squalene-2,3-epoxide
5.4.99.7
5.4.99.8
24,25-Dihydro-lanosterol
1.3.1.72
Lanosterol
Cycloartenol
1.14.14.154
1.14.15.36
4,4-Dimethyl-cholesta-8,14,24-trienol
SMT1
1.3.1.70
4,4-Dimethyl-cholesta-8-en-3β-ol
1.3.1.72
14-Demethyl-lanosterol
24-Methylene-cycloartanol
1.14.18.9
1.14.18.10
4-Methylzymosterol-carboxylate
3β-Hydroxy-4β,14α-dimethyl-9β,19-cyclo-5α-ergost-24(24(1))-en-4α-carboxylate
1.1.1.170
1.1.1.418
3-Keto-4-methyl-zymosterol
Cycloeucalenone
1.1.1.270
1.1.1.270
4-Methylzymosterol
Cycloeucalenol
1.14.18.9
5.5.1.9
4α-Carboxy-5α-cholesta-8,24-dien-3β-ol
Obtusifoliol
1.1.1.170
CYP51G1
Zymosterone
5α-8,14-Sterol
FK
1.1.1.270
4α-Methylfecosterol
HYD1
Cholesta-8-en-3β-ol
1.3.1.72
Zymosterol
24-Methylene-lophenol
SMT2
24-Ethylidene-lophenol
ERG6
1.14.18.11
1.14.18.11
3β-Hydroxyergosta-7,24(24(1))-dien-4α-carboxylate
4α-Carboxy-stigmasta-7,24(24(1))-dien-3β-ol
5.3.3.5
5.3.3.5
1.1.1.418
1.1.1.418
Fecosterol
Episterone
Avenastenone
ERG2
1.1.1.270
1.1.1.270
Lathosterol
1.3.1.72
Cholesta-7,24-dien-3β-ol
Episterol
Episterol
Δ7-Avenasterol
1.14.19.20
ERG3
STE1
STE1
Vitamin D3
7-Dehydrocholesterol
1.3.1.72
7-Dehydro-desmosterol
5-Dehydroepisterol
5-Dehydroepisterol
5-Dehydroavenasterol
hν
1.14.14.24
1.14.15.22
1.3.1.21
ERG5
DWF5
DWF5
Cholesterol ester
3.1.1.13
1.3.1.72
Desmosterol
5,7,22,24(28)-Ergosta-tetraenol
24-Methylenecholesterol
Isofucosterol
Calcidiol
2.3.1.26
Cholesterol
ERG4
DWF1
DWF1
DWF1
1.14.15.18
1.14.15.22
2.3.1.73
Steroid hormone biosynthesis
24-epi-Campesterol
Ergosterol
Campesterol
Sitosterol
Calcitriol
Steroid degradation
hν
CYP710A
CYP710A
1.14.15.16
1.14.15.16
Brassicasterol
Stigmasterol
Calcitetrol
Secalciferol
Primary bile acid biosynthesis
Vitamin D2
Brassinosteroid biosynthesis
Phytosterol
Data on KEGG graph
Rendered by Pathview

examples of upregulated pathways

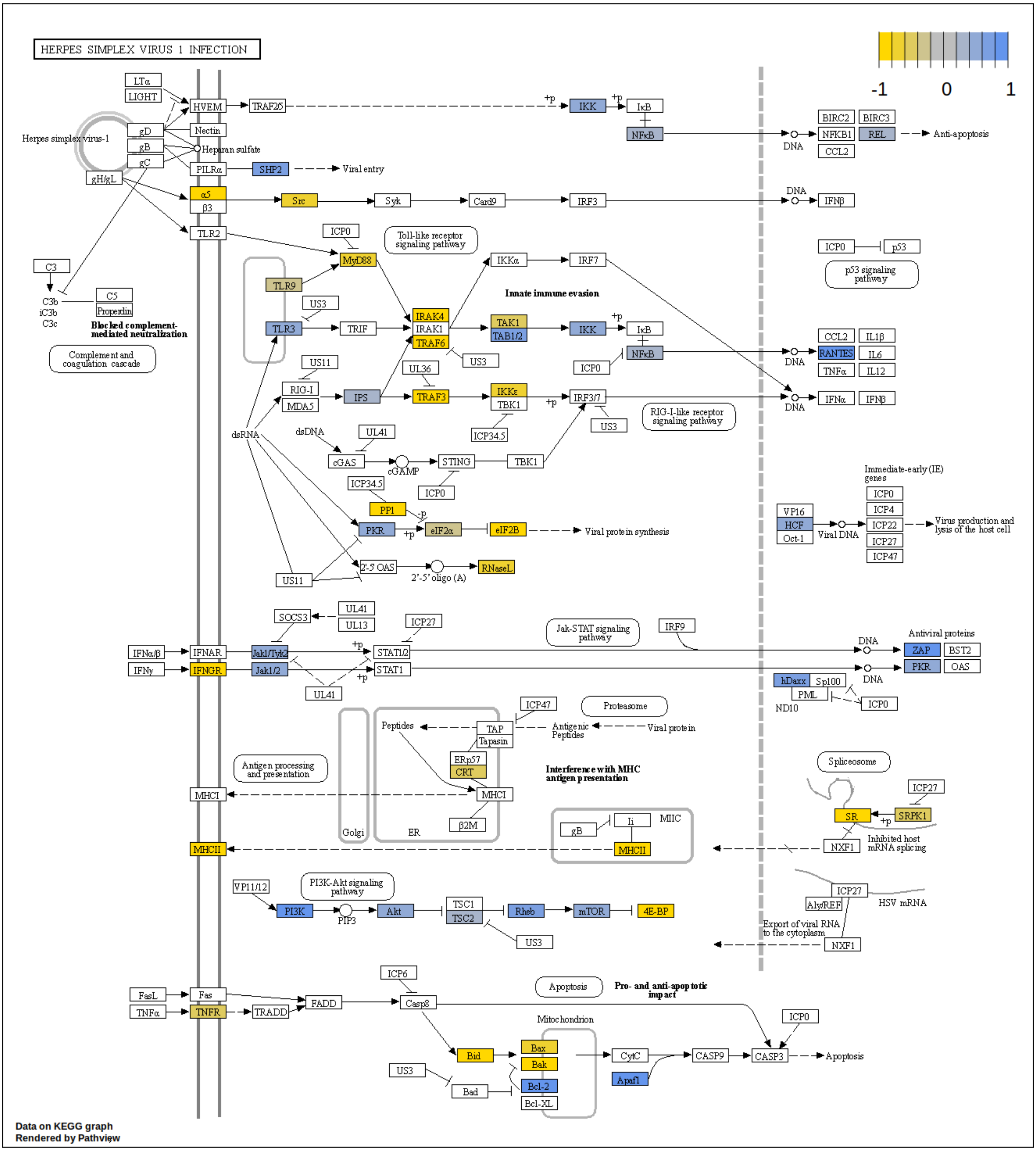

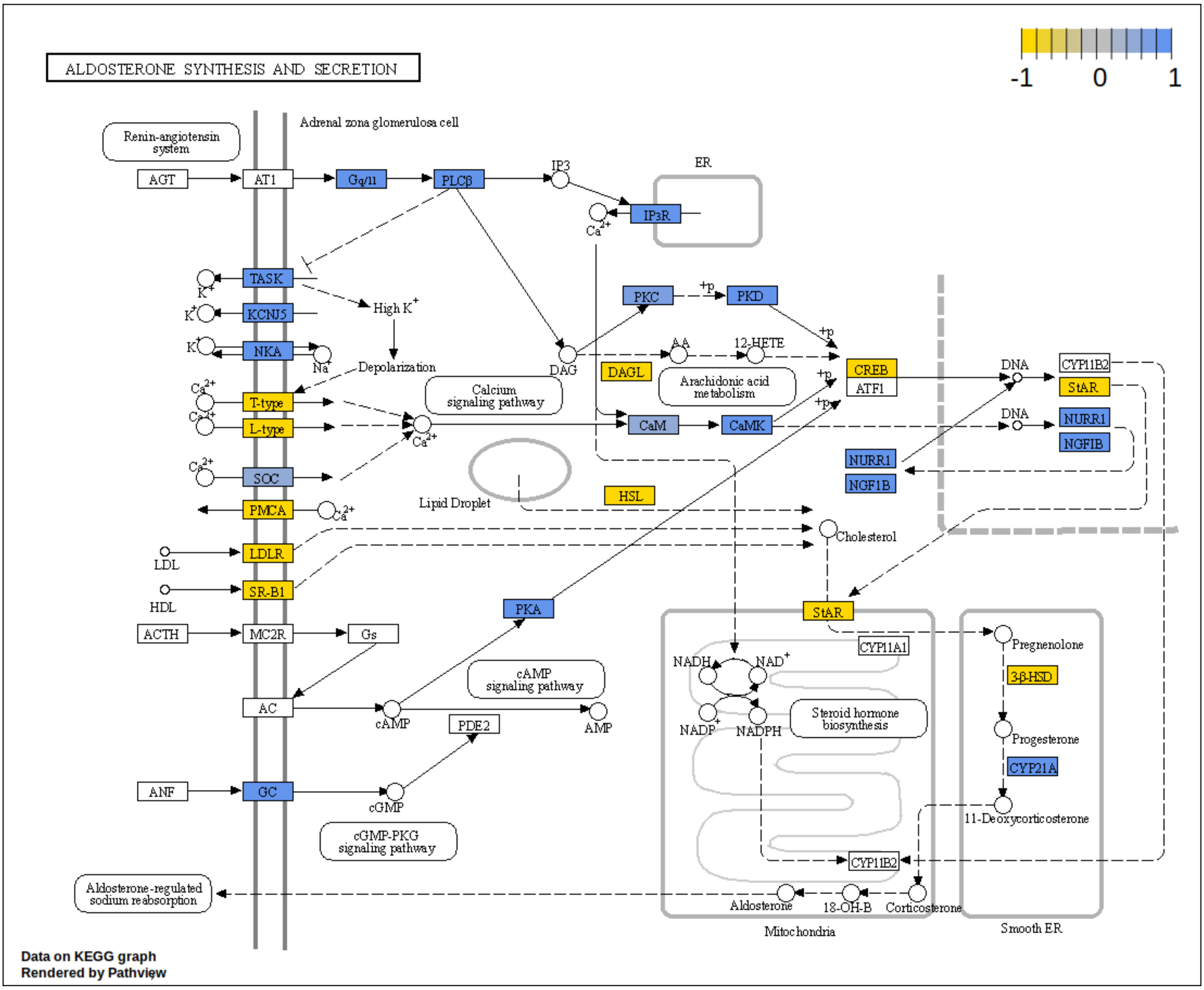
ALDOSTERONE SYNTHESIS AND SECRETION
-1
0
1
Adrenal zona glomerulosa cell
Renin-angiotensin system
AGT
AT1
Gq/11
PLCβ
IP3
ER
IP3R
Ca2+
TASK
KCNJ5
NKA
K+
Na+
High K+
Depolarization
T-type
L-type
SOC
PMCA
LDLR
SR-B1
LDL
HDL
ACTH
MC2R
Gs
AC
cAMP
AMP
PDE2
ANF
GC
cGMP
cGMP-PKG signaling pathway
cAMP signaling pathway
Calcium signaling pathway
PKC
PKD
+p
DAG
DAGL
AA
12-HETE
Arachidonic acid metabolism
CREB
ATF1
DNA
CYP11B2
StAR
NURR1
NGFIB
CaM
CaMK
PKA
HSL
Lipid Droplet
Cholesterol
CYP11A1
NADH
NAD+
NADP+
NADPH
Steroid hormone biosynthesis
Pregnenolone
3β-HSD
Progesterone
CYP21A
11-Deoxycorticosterone
Corticosterone
18-OH-B
Aldosterone
Aldosterone-regulated sodium reabsorption
Mitochondria
Smooth ER
Data on KEGG graph
Rendered by Pathview

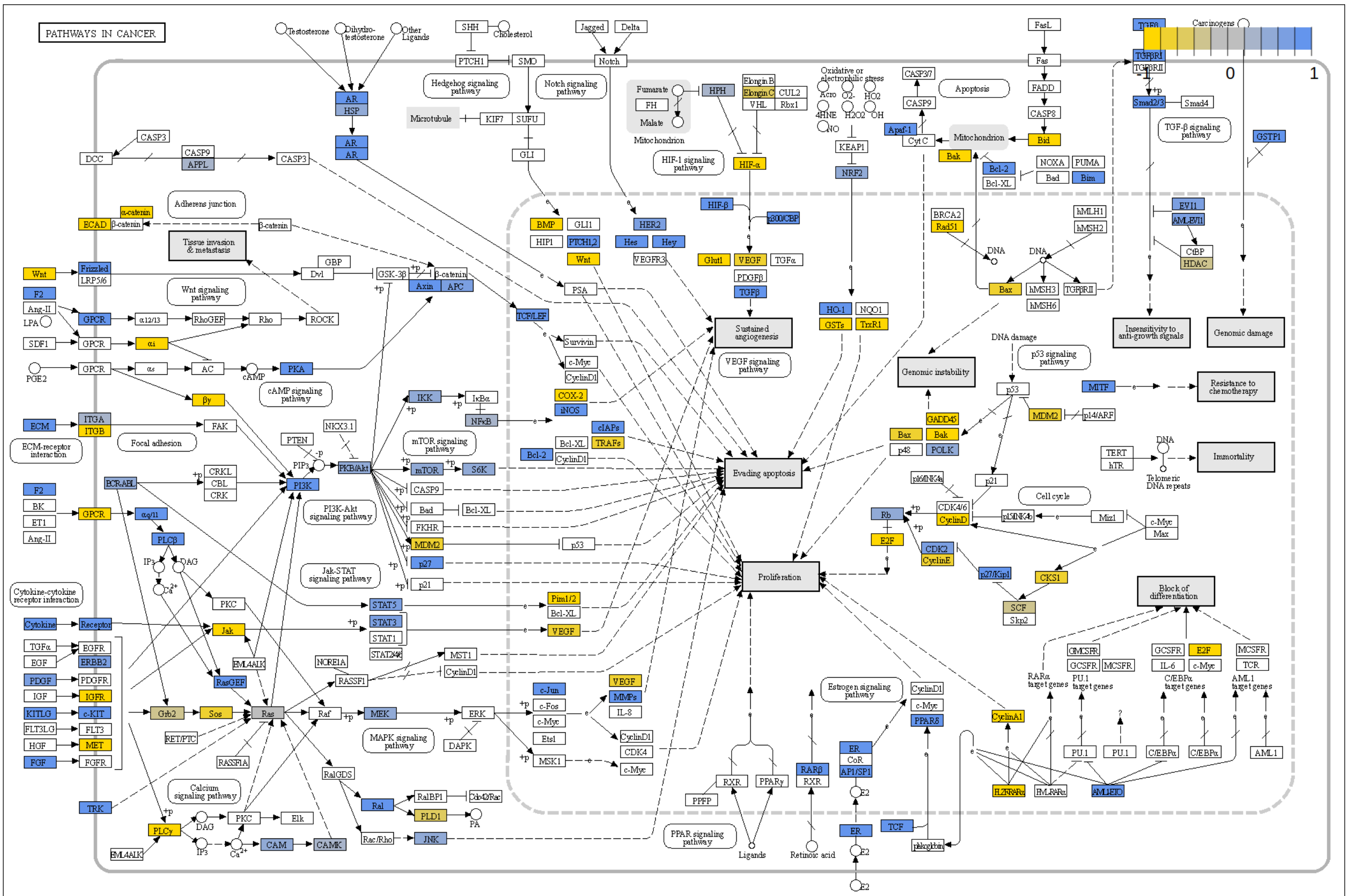

Data on KEGG graph
Rendered by Pathview

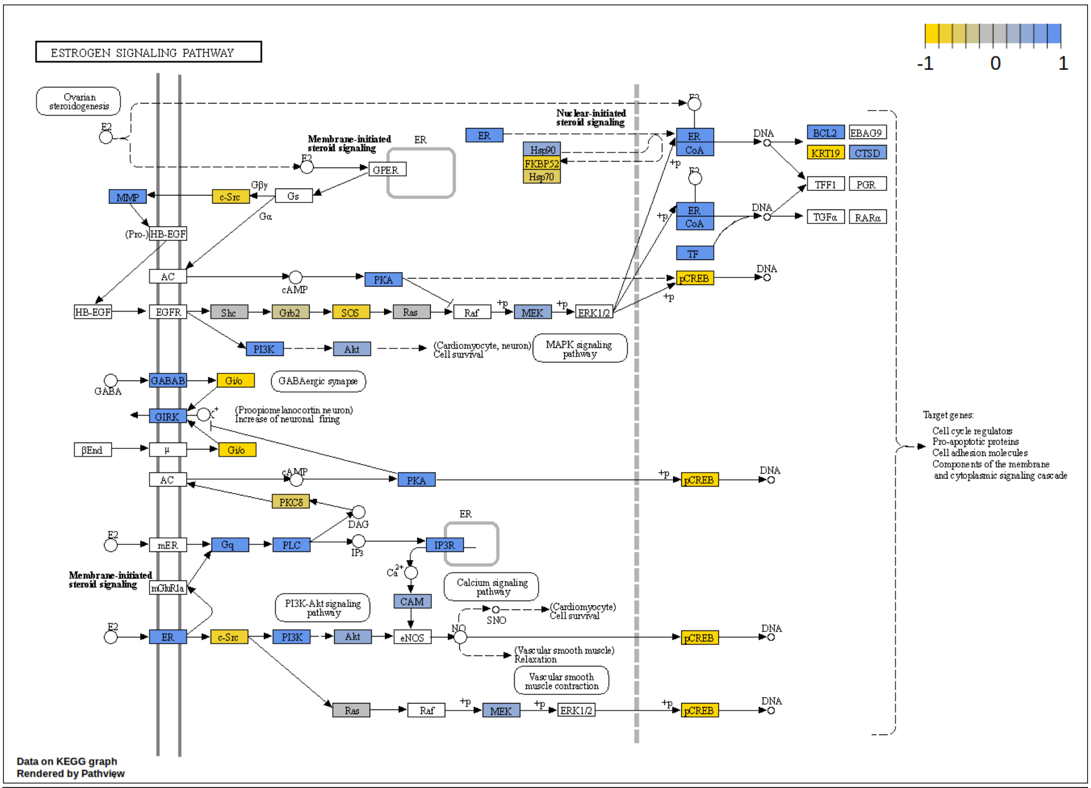

ESTROGEN SIGNALING PATHWAY
-1 0 1
Membrane-initiated steroid signaling
Nuclear-initiated steroid signaling
Ovarian steroidogenesis
MAPK signaling pathway
GABAergic synapse
Calcium signaling pathway
PI3K-Akt signaling pathway
Vascular smooth muscle contraction
Target genes:
Cell cycle regulators
Pro-apoptotic proteins
Cell adhesion molecules
Components of the membrane and cytoplasmic signaling cascade
Data on KEGG graph
Rendered by Pathview

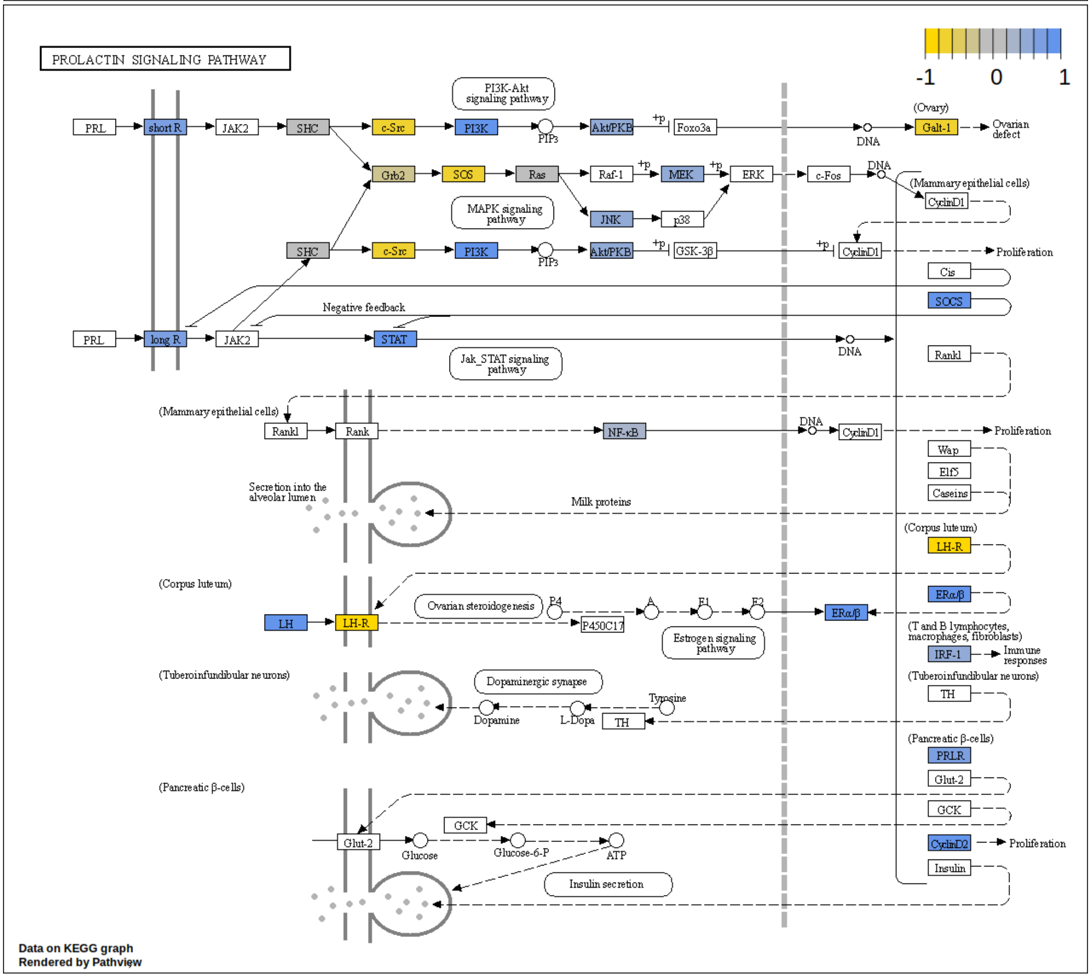

PROLACTIN SIGNALING PATHWAY
-1 0 1
PI3K-Akt signaling pathway
MAPK signaling pathway
Jak_STAT signaling pathway
Negative feedback
Ovarian steroidogenesis
Estrogen signaling pathway
Dopaminergic synapse
Insulin secretion
Milk proteins
Proliferation
Data on KEGG graph
Rendered by Pathview
